# Multiple mechanisms lead to loss-of-function effects of pathogenic *SARS2* variants

**DOI:** 10.1242/dmm.052690

**Published:** 2026-07-24

**Authors:** Christina Del Greco, Stephanie M. Laureano Figueroa, Anthony Antonellis

**Affiliations:** ^1^ Department of Human Genetics, University of Michigan Medical School, Ann Arbor, MI 48109, USA; ^2^ Medical Scientist Training Program, University of Michigan Medical School, Ann Arbor, MI 48109, USA; ^3^ Department of Neurology, University of Michigan Medical School, Ann Arbor, MI 48109, USA

**Keywords:** Mitochondrial tRNA synthetase, HUPRA syndrome, Progressive spastic paresis, Aminoacyl-tRNA synthetase, Mendelian disease

## Abstract

Seryl-tRNA synthetase 2 (*SARS2*) encodes the enzyme responsible for charging tRNA with serine in the mitochondria. *SARS2* has been associated with a spectrum of recessive diseases including HUPRA syndrome and progressive spastic paresis. Previous studies showed that pathogenic *SARS2* variants cause decreased tRNA charging; however, the mechanism by which specific variants lead to distinct recessive phenotypes has not been defined. To address this lack of knowledge, we studied an allelic series of 11 pathogenic *SARS2* variants for differential effects on mitochondrial function. These efforts revealed compelling variant-dependent effects on oxygen consumption that will be useful for genotype–phenotype correlations. Interestingly, certain variants (including the most commonly detected pathogenic *SARS2* variant, R402H) did not affect mitochondrial function in our model system. Computational and functional studies revealed that two missense variants in exon 13 (D390G and R402H) reduce exon inclusion, suggesting loss-of-function effects via impaired transcript processing. Overall, this study expands our understanding of *SARS2* biology, reveals differential effects that pathogenic variants have on *SARS2* function, and provides the foundation for defining the clinical heterogeneity of patient phenotypes.

## Teaser

**Summary:** Pathogenic *SARS2* variants cause a spectrum of recessive disease phenotypes. Our functional studies revealed that pathogenic missense variants impact different aspects of gene function and that the degree of impairment is consistent with clinical severity.

## INTRODUCTION

Aminoacyl-tRNA synthetases (ARSs) are essential enzymes that ligate tRNA to cognate amino acids in the cytoplasm and mitochondria. The human nuclear genome encodes 37 ARSs, 17 of which localize exclusively to the mitochondria and are essential for the translation of the 13 mitochondrial-encoded proteins (Del Greco and Antonellis, 2022). These mitochondrial-encoded proteins make up components of the oxidative phosphorylation machinery and are required for effective generation of ATP by aerobic respiration (Nicholls and Gustafsson, 2018).

Importantly, all 17 mitochondrial ARSs are associated with human recessive diseases (Del Greco and Antonellis, 2022). The clinical phenotypes often affect tissues with high energy demands such as the central nervous system, cardiovascular system and musculoskeletal system (Craven et al., 2017). Although dysfunction of any given mitochondrial ARS should largely impact oxidative phosphorylation function by disrupting the translation of mitochondrial-encoded proteins, diseases associated with mitochondrial ARSs display vast clinical heterogeneity (Sissler et al., 2017). While many patients with mitochondrial ARS-related diseases present with phenotypes including encephalopathies and cardiomyopathies consistent with commonly affected tissues in mitochondrial disease, other patients present with conditions like Perrault syndrome, which affects the ovaries and leads to sensorineural hearing loss (Boczonadi et al., 2018; Gotz et al., 2011; Pierce et al., 2011). A major question in the field that needs to be addressed is by what mechanism does impairment in mitochondrial ARS function lead to the observed clinical heterogeneity.

A particularly compelling example of a mitochondrial ARS associated with a distinct range of clinical phenotypes is mitochondrial seryl-tRNA synthetase [*SARS2*; Online Mendelian Inheritance in Man (OMIM) 612804], which encodes the enzyme that ligates serine to tRNA in the mitochondria. The first reports of *SARS2*-associated disease described patients with a multisystem condition that includes hyperuricemia, pulmonary hypertension, renal failure in infancy and alkalosis (HUPRA syndrome) (Belostotsky et al., 2011; Rivera et al., 2013). Since then, there have been reports of patients with *SARS2*-related recessive disease that present with an isolated progressive spastic paresis (PSP) (Linnankivi et al., 2016). PSP is characterized by limb spasticity in the lower extremities that progresses to the upper extremities, and can include cognitive delay and cerebellar atrophy. Previous studies have shown that pathogenic *SARS2* variants cause defects in tRNA charging, specifically in ligating serine to the tRNA^Ser^(AGY) isoacceptor (Yu et al., 2022), consistent with the hypothesis that impaired *SARS2* function will diminish the population of charged tRNAs and inhibit mitochondrial protein translation. However, these studies have focused on a small number of alleles, and, to date, no study has assessed the functional consequences of all known pathogenic *SARS2* variants toward determining the mechanism by which different *SARS2* variants lead to distinct clinical phenotypes.

In this study, we developed a human cell complementation model to define (1) the cellular effects of *SARS2* loss; and (2) the functional consequences of an allelic series of 11 pathogenic *SARS2* variants, including alleles associated with HUPRA syndrome and alleles associated with PSP. We employed Hap1 cells to knock out the endogenous *SARS2* gene after introducing a second, doxycycline-inducible wild-type copy to support cell growth in the absence of endogenous *SARS2*. First, we leveraged this model to assess for transcriptional changes and effects on mitochondrial oxygen consumption that result from ablating *SARS2* expression (i.e. in the absence of doxycycline). Next, we developed lentiviruses to individually introduce an allelic series of pathogenic *SARS2* variants and to investigate the ability of each allele to support mitochondrial oxygen consumption; these efforts revealed a range of loss-of-function effects for eight of the 11 variants studied. Interestingly, certain variants (including the most commonly detected pathogenic variant, R402H) showed no significant deficit in oxygen consumption compared to wild-type *SARS2*. Computational and functional studies revealed that two missense variants in exon 13 impact proper splicing of *SARS2*, resulting in loss-of-function effects via impaired transcript processing. In summary, this study provides insights into the role of *SARS2* in cellular and mitochondrial function, identifies functional differences among a large panel of pathogenic *SARS2* variants, and expands our understanding of the various mechanisms by which pathogenic *SARS2* variants lead to loss-of-function effects.

## RESULTS

### An allelic series of pathogenic *SARS2* variants

**Fig. 1.**
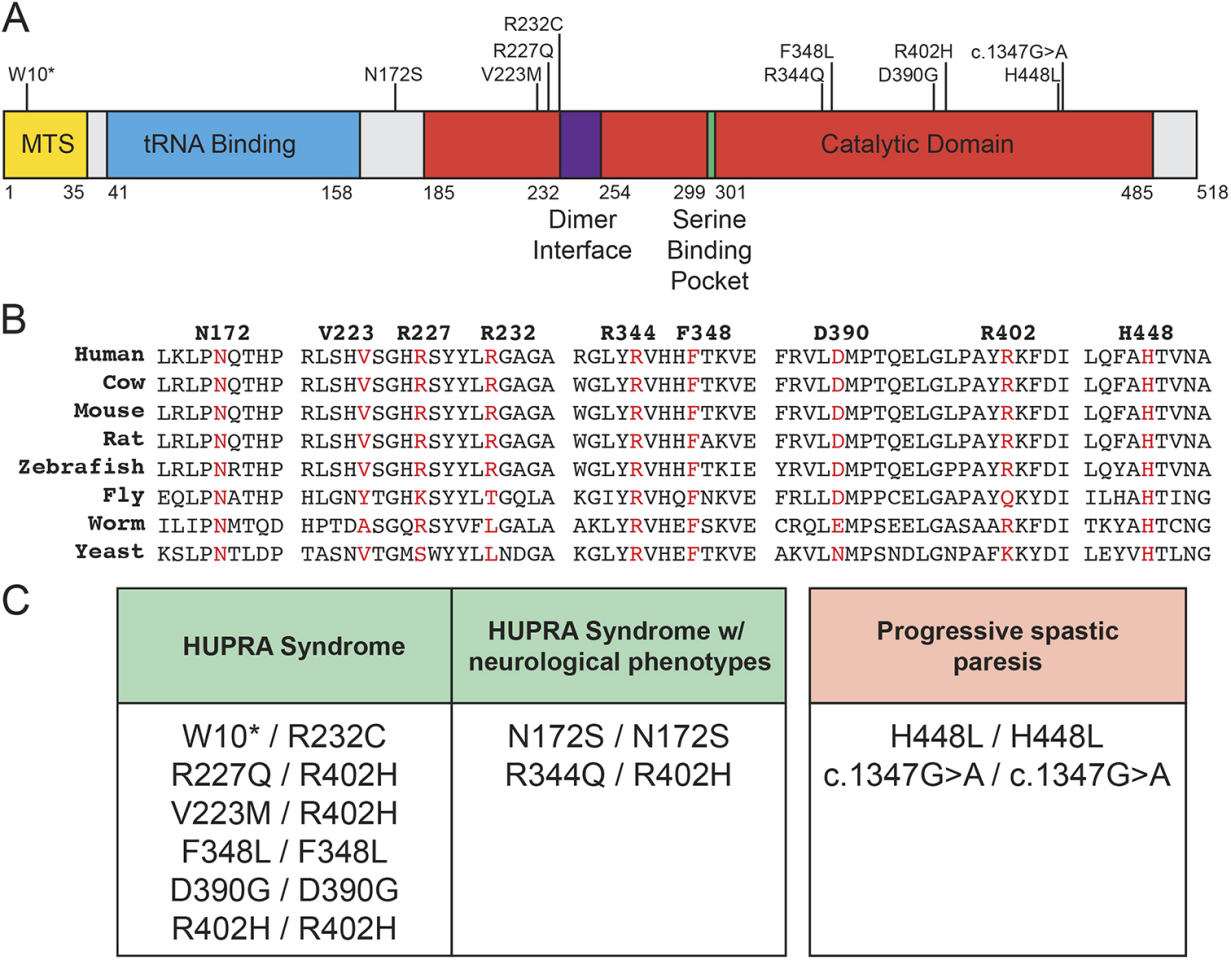
**Panel of pathogenic *SARS2* variants for assessment.** (A) Protein domain structure of SARS2 protein with pathogenic variants labeled. MTS, mitochondrial targeting sequence. Numbers at the bottom of the diagram indicate amino acid position. (B) Multiple-species protein alignments of SARS2 showing the position of missense variants. Human amino acid positions are indicated at the top of each sequence, and the amino acid of interest is indicated in red. (C) Table of clinical phenotypes and the associated pathogenic *SARS2* variants.

To interrogate correlations between genotypes and phenotypes observed in *SARS2-*mediated disease, we selected an allelic series of pathogenic *SARS2* variants to perform analyses on protein expression and mitochondrial function. We selected 11 variants in total: nine missense changes, one nonsense change and one splice-modifying variant. The majority of the variants tested fall within the catalytic domain of SARS2, with the exceptions of W10*, which truncates the protein in the mitochondrial targeting sequence, and N172S, which falls between the tRNA binding and catalytic domains (Fig. 1A). Interestingly, R232C also overlaps with the beginning of the dimerization domain (Fig. 1A). Multiple species alignments of each variant indicate that many impacted residues are highly conserved among species (Fig. 1B). Population frequency data from gnomAD (Chen et al., 2024) indicate that these pathogenic variants are rare in the general population; none of the variants are found in the homozygous state (Table 1). Taken together, these data support the pathogenicity of the variants selected for study. In total, we selected six variants associated with HUPRA syndrome, two variants associated with PSP and three variants associated with clinical phenotypes that overlap both conditions (Fig. 1C).

**Table 1.** Pathogenic *SARS2* variants and corresponding gnomAD variant frequencies

Variant	Source	gnomAD frequency	Homozygotes
Trp10Ter (W10*)	ClinVar Accession VCV000215114.4	0	0
Asn172Ser (N172S)	Goknar et al., 2022	3/1,614,172 (1.86×10^−6^)	0
Val223Met (V223M)	Zhou et al., 2021	6/1,613,994 (3.72×10^−6^)	0
Arg227Gln (R227Q)	Yang et al., 2022	43/1,614,096 (2.66×10^−5^)	0
Arg232Cys (R232C)	ClinVar Accession VCV000432690.6	35/1,614,076 (2.17×10^−5^)	0
Arg344Gln (R344Q)	Colin et al., 2021	10/1,610,682 (6.21×10^−6^)	0
Phe348Leu (F348L)	ClinVar Accession VCV000242874.1	0	0
Asp390Gly (D390G)	Belostotsky et al., 2011	0	0
Arg402His (R402H)	Colin et al., 2021; Rivera et al., 2013; Roux et al., 2021; Yang et al., 2022; Zhou et al., 2021	100/1,613,932 (6.20×10^−5^)	0
His448Leu (H448L)	Souza et al., 2017	0	0
c.1347G>A (T449=)	Linnankivi et al., 2016	28/1,613,470 (1.74×10^−5^)	0

### A human cell complementation model to study *SARS2*

**Fig. 2.**
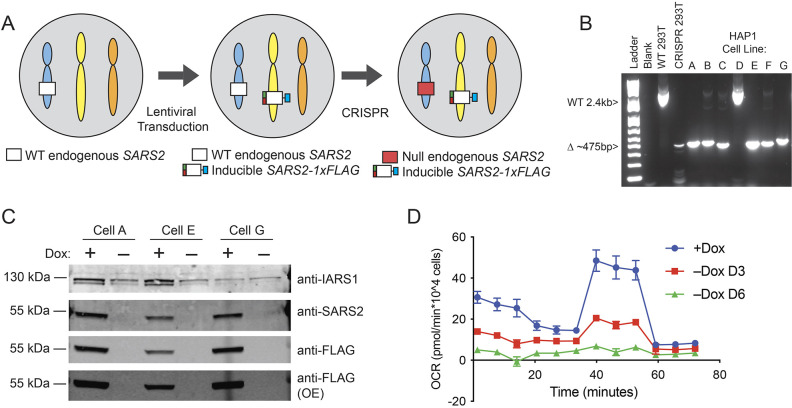
**Generation of Δ*SARS2+SARS2-1xFLAG* Hap1 cells.** (A) Cartoon outline of the combined lentiviral transduction and CRISPR editing approach to generate Δ*SARS2+SARS2-1xFLAG* cells. (B) Gel image of PCR genotyping around the expected endogenous *SARS2* deletion; the expected size of an unedited PCR product is 2.4 kb, and the expected size of an edited PCR product is 475 bp. (C) Western blots of SARS2 and FLAG expression in selected clonal cell lines demonstrating protein expression in the presence and absence of doxycycline after 5 days. Representative of three western blots. (D) Seahorse mitochondrial stress test on Δ*SARS2+SARS2-1xFLAG* cells either treated with 15 ng/ml doxycycline or grown without doxycycline for 3 or 6 days. Oxygen consumption data are normalized by cell count. *n*=3 per condition. OCR, oxygen consumption rate; WT, wild type.

To study the effects of *SARS2* loss in human cells and to study the functional consequences of pathogenic *SARS2* variants, we generated a haploid (Hap1) cell model containing (1) a doxycycline-inducible transgene that includes a C-terminal FLAG-tagged *SARS2* open reading frame with protospacer adjacent motif (PAM) site mutations to protect the transgene from cutting by Cas9; and (2) a CRISPR/Cas9-induced 2 kb deletion at the endogenous *SARS2* locus (Fig. 2A). Once established, the consequences of *SARS2* depletion could be studied by removing doxycycline from cell culture media. Additionally, the functional consequences of *SARS2* variants could be studied by introducing variant-specific expression constructs followed by removal of doxycycline.

To generate this model, the *SARS2-1xFLAG* transgene was cloned into the pCW57.1 lentiviral vector under the control of a doxycycline-inducible promoter. Parental Hap1 cells were transduced with the *SARS2+1xFLAG/pCW57.1* lentivirus. After selection with puromycin, transduced cells were treated with doxycycline and transfected with two all-in-one CRISPR sgRNA plasmids (containing GFP markers) to introduce a 2 kb deletion in the catalytic domain of the endogenous locus (genomic coordinates GRCh37: chr19:39,406,680-39,408,647); while this deletion remains in-frame, it deletes amino acids 322-448, which span almost four complete exons encoding part of the catalytic domain of the SARS2 protein. Cells were next subjected to flow sorting for GFP-positive cells to select for sgRNA uptake and then plated as individual cells in 96-well plates. Six surviving colonies were collected, and a portion of the cells were used to extract genomic DNA. PCR analysis with primers spanning the predicted deletion was used to select properly mutated clones, which revealed three promising clones (A, E and G in Fig. 2B). These three clonal lines contain only the ‘deletion’ PCR product and no evidence of the ‘wild-type’ PCR product. DNA from these PCR reactions was submitted for sequencing to confirm the deletion (Fig. S1); lines A, E and G were all validated, and subsequent experiments were performed on clone A with certain validation studies performed on clone E.

To confirm that this cell model impairs SARS2 protein expression, the three cell lines were grown without doxycycline for 5 days, then collected to extract total cellular protein. Western blot analysis indicated that all three cell lines grown without doxycycline had undetectable levels of both SARS2 protein and FLAG-tag protein (Fig. 2C), demonstrating that the removal of doxycycline halts the expression of SARS2-1xFLAG and that endogenous SARS2 is no longer expressed (Fig. 2C). These cells are referred to as Δ*SARS2+SARS2-1xFLAG*.

Because SARS2 is essential for mitochondrial protein translation, we predicted that the loss of *SARS2* expression would lead to defects in mitochondrial function. To assess this, we performed Seahorse mitochondrial stress test assays on Δ*SARS2*+*SARS2*-*1xFLAG* cells to look for changes in oxygen consumption. Cell lines A and E were grown with doxycycline or without doxycycline for 3 or 6 days and then subjected to an assessment of oxygen consumption via Seahorse assays. These studies revealed that, by 3 days after removal of doxycycline, cellular oxygen consumption was significantly decreased (red line in Fig. 2D), and that, by 6 days after removal of doxycycline, cellular oxygen consumption was almost exclusively non-mitochondrial (green line in Fig. 2D; Fig. S2), indicating severe defects in mitochondrial function associated with the loss of *SARS2* expression. Thus, this cellular model will be informative for studies on the effects of *SARS2* loss on cellular functions and for the functional consequences of pathogenic *SARS2* variants.

### Loss of *SARS2* in Hap1 cells results in activation of cell stress pathways

**Fig. 3.**
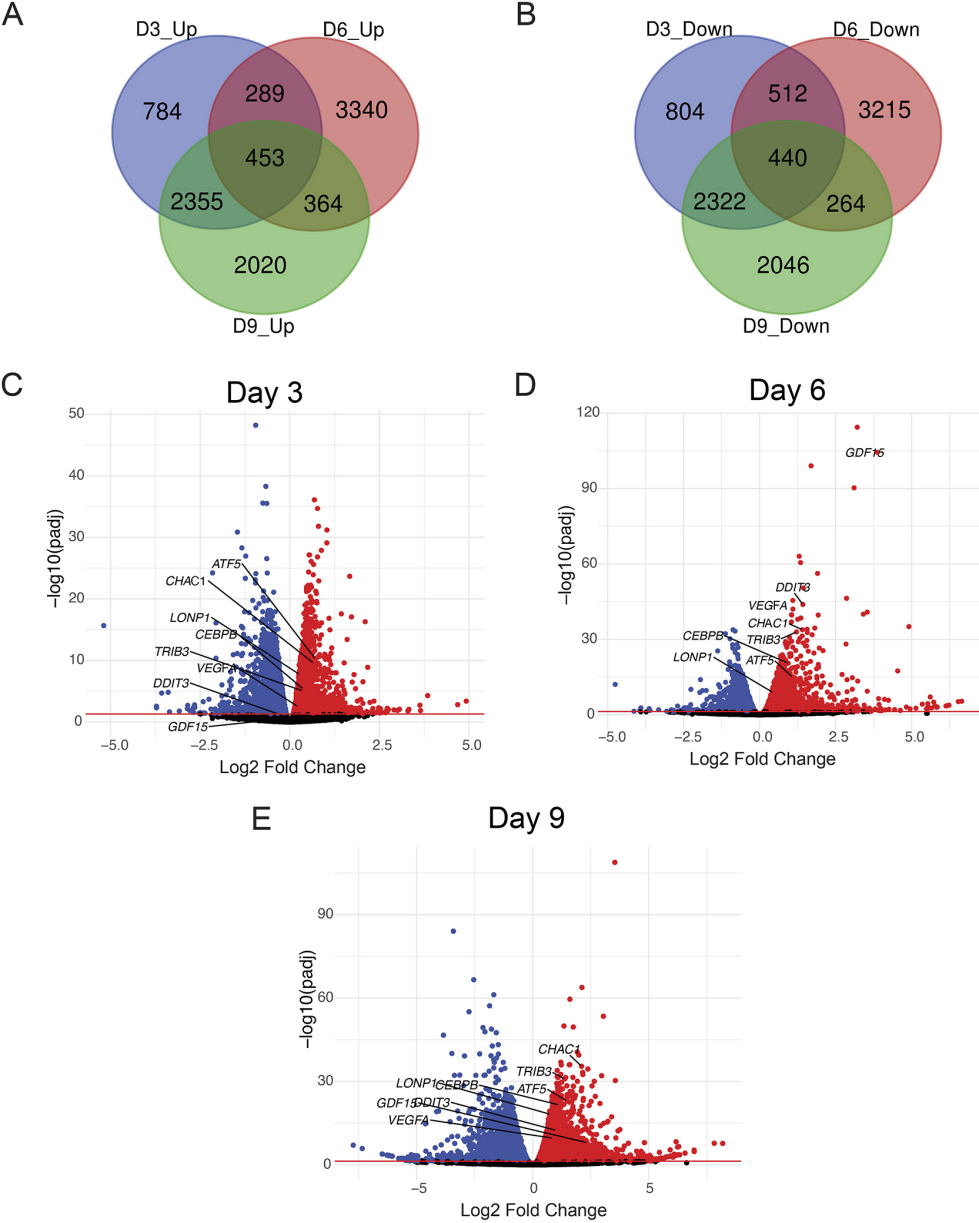
**Loss of SARS2 expression in Δ*SARS2+SARS2-1xFLAG* cells identifies differentially expressed genes.** (A,B) Venn diagrams indicating numbers of differentially expressed genes that are significantly [adjusted *P*-value (padj)<0.05] upregulated (A) or downregulated (B) in ΔSARS2+SARS2-1xFLAG cells at 3, 6 or 9 days without doxycycline compared to those in doxycycline-treated controls. (C-E) Volcano plots of differentially expressed genes at days 3 (C), 6 (D) and 9 (E) compared to those in doxycycline-treated controls. The red line indicates statistically significant genes (padj<0.05); genes in blue are downregulated and genes in red are upregulated. Highlighted genes include *ATF5*, *CEBPB*, *CHAC1*, *DDIT3*, *LONP1*, *TRIB3* and *VEGFA*, all of which show significant gene expression changes at one or more time point. Three independently isolated RNA samples per condition.

Because *SARS2* is an essential gene that is required for mitochondrial translation, we would expect that complete loss of *SARS2* expression would be lethal and that the cellular response to the depletion of *SARS2* would include signs of mitochondrial dysfunction. To assess for any transcriptional changes associated with the loss of *SARS2* expression, Δ*SARS2+SARS2-1xFLAG* cells were grown in the presence or absence of doxycycline for 3, 6 or 9 days before being harvested for RNA isolation and RNA-sequencing (RNA-seq) analysis. Sequencing data were analyzed for significantly differentially expressed genes [adjusted *P*-value (padj)<0.05] at each time point compared to those in doxycycline-positive controls. These analyses revealed 453 genes upregulated at all three time points and 440 genes downregulated at all three time points, relative to those in doxycycline-positive controls (Fig. 3A,B). Volcano plots of differentially expressed genes at each time point compared to those in doxycycline-positive cells were generated to show the distributions of upregulated and downregulated genes (Fig. 3C-E). To assess for potential doxycycline effects, we assessed for differentially expressed genes between unmodified Hap1 cells and Hap1 cells treated with doxycycline. To assess for potential effects of doxycycline treatment and/or transgene insertion on the transcriptome that could confound our comparisons between doxycycline-treated and untreated transgene-modified cells, we assessed for differentially expressed genes between Δ*SARS2+SARS2-1xFLAG* cells treated with doxycycline and unmodified Hap1 cells treated with doxycycline (Fig. S3). Broadly, we found that the depletion of *SARS2* expression greatly impacted the cellular transcriptome, as would be expected with the loss of an essential gene.

**Fig. 4.**
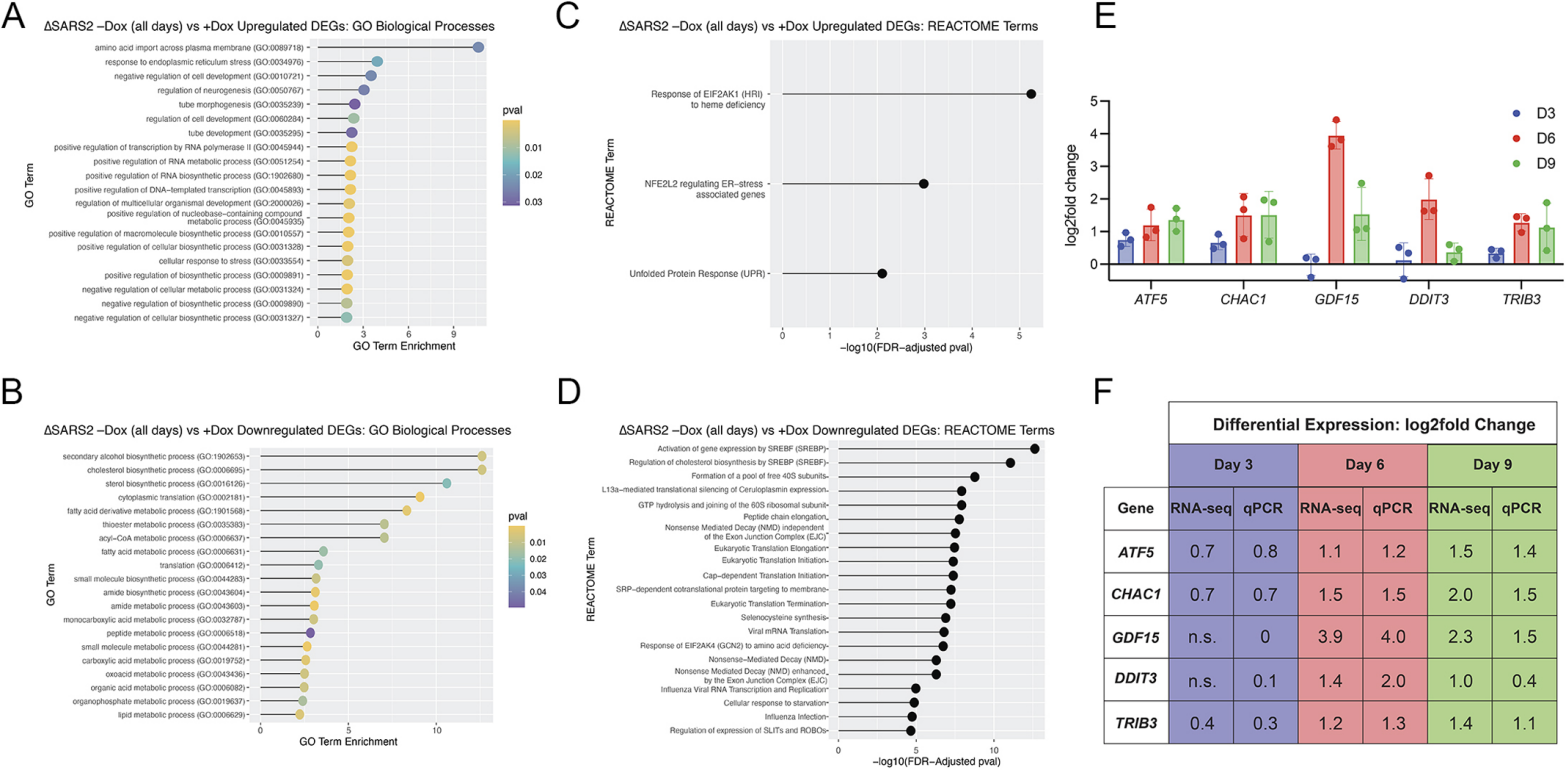
**Differentially expressed genes include many cellular stress-associated genes and pathways.** (A,B) GO analyses of upregulated (A) and downregulated (B) genes that overlap at all three assessed time points. GO Terms for Biological Processes were graphed by enrichment score, and colored dots indicate the *P*-value. (C,D) REACTOME analyses of upregulated (C) and downregulated (D) genes that overlap at all three assessed time points. REACTOME terms were graphed by the –log10 of the FDR-padj; only terms with a padj<0.05 were selected. (E) qPCR validation of target genes identified as being both significantly upregulated in cells and associated with cellular stress pathways. Fold changes were calculated using the 2^-ΔΔCt^ method using *REEP5* as the control gene, and resulting fold changes were log2 transformed. Table below compares the differential expression values determined from RNA-seq to those determined by qPCR. *n*=3 per condition, per gene tested. D, day; DEG, differentially expressed gene; FDR, false discovery rate; GO, Gene Ontology; pval, *P*-value; qPCR, quantitative PCR, RNA-seq, RNA sequencing.

Gene Ontology (GO) and REACTOME analyses (Ashburner et al., 2000; Fabregat et al., 2017; Gene Ontology Consortium et al., 2023) were performed on differentially expressed genes to identify major biological pathways that are upregulated and downregulated in response to *SARS2* depletion (Fig. 4A-D; Fig. S4). The top 20 terms (or all terms, if the number of significant terms was less than 20) were identified for analysis. GO and REACTOME analyses performed on genes that were differentially downregulated at all three time points relative to those in doxycycline-treated cells identified pathways involved in mitochondrial function [e.g. secondary alcohol biosynthesis (12.61-fold enrichment, *P*=6.96×10^−3^) and fatty acid metabolism (9.08-fold enrichment, *P*=2.83×10^−8^)] and protein translation pathways (Fig. 4B,D). These findings further validated our model and were consistent with severe mitochondrial dysfunction caused by loss of an essential mitochondrial gene.

We also performed the above analyses on genes that were differentially upregulated at all three time points relative to those in doxycycline-treated cells. Notably, both GO and REACTOME analyses revealed the upregulation of cellular stress pathways upon the loss of *SARS2*. This included the upregulation of the heme-regulated inhibitor (HRI) arm of the integrated stress response (ISR) [false discovery rate (FDR)-padj=5.68×10^−6^], endoplasmic reticulum (ER) stress-associated genes (FDR-padj=0.001) and the unfolded protein response (UPR) (FDR-padj=0.008) (Fig. 4A,C). We highlight eight of these genes (*ATF5*, *CHAC1*, *LONP1*, *CEBPB*, *TRIB3*, *VEGFA*, *DDIT3* and *GDF15*) that represent all three of the stress pathways identified: the ISR (*ATF5*, *CHAC1*, *CEBPB*, *TRIB3*, *VEGFA*, and *GDF15*), ER stress (*ATF5* and *DDIT3*) and the UPR (*LONP1*). All eight genes were consistently upregulated (or showed no change) across all three time points in the absence of *SARS2* expression (Fig. 3C-E) and were never downregulated in the absence of *SARS2* expression at any time point (Fig. 3C-E). To further support these findings, we compiled a manually curated list of 103 genes associated with a range of stress pathways via literature review (Condon et al., 2021; Fiorese et al., 2016; Kress et al., 2023; Neill and Masson, 2023; Quiros et al., 2017; Teske et al., 2013; Torrence et al., 2021; Zhao et al., 2002). Assessment of this curated list in our RNA-seq data also supported the observation that stress-associated genes were upregulated in cells lacking *SARS2* expression (Fig. S5). Of the genes assessed, ATF4-target genes showed the highest levels of consistent upregulation, followed by genes in the UPR and ER stress pathways (Fig. S5). We also noted the upregulation of genes in the mTORC1 pathway, suggesting another stress pathway that may be activated in these cells (Fig. S5). Finally, quantitative PCR (qPCR) analysis of selected stress-related genes identified in the above studies (*ATF5*, *CHAC1*, *GDF15*, *DDIT3* and *TRIB3*) validated our original RNA-seq data (Fig. 4E,F). In summary, the loss of *SARS2* in our cell model was associated with decreased expression of genes implicated in mitochondrial function and protein translation, and increased expression of genes implicated in cellular stress responses. These data further validated our model and raised important questions about the downstream effects of impaired *SARS2* function.

### Pathogenic *SARS2* variants show differential effects on SARS2 protein levels

After confirming that our cell model can be used effectively to study the effects of *SARS2* depletion, we sought to use it as a complementation system to assess the impact of the full panel of pathogenic *SARS2* variants on both protein expression and mitochondrial function. To do so, a *SARS2* open reading frame in an entry vector containing a C-terminal myc tag and the intron 14 sequence (referred to as *SARS2+int14-myc*) was generated to study each pathogenic variant. Importantly, *SARS2* intron 14 was incorporated into the expression construct to include the c.1347G>A (T449=) variant, which impairs splicing and is one of only two variants thus far associated with PSP (Linnankivi et al., 2016). Each variant of interest was mutagenized into the *SARS2+int14-myc* construct and then cloned into the pLenti PGK Hygro lentiviral vector and submitted for lentiviral generation.

**Fig. 5.**
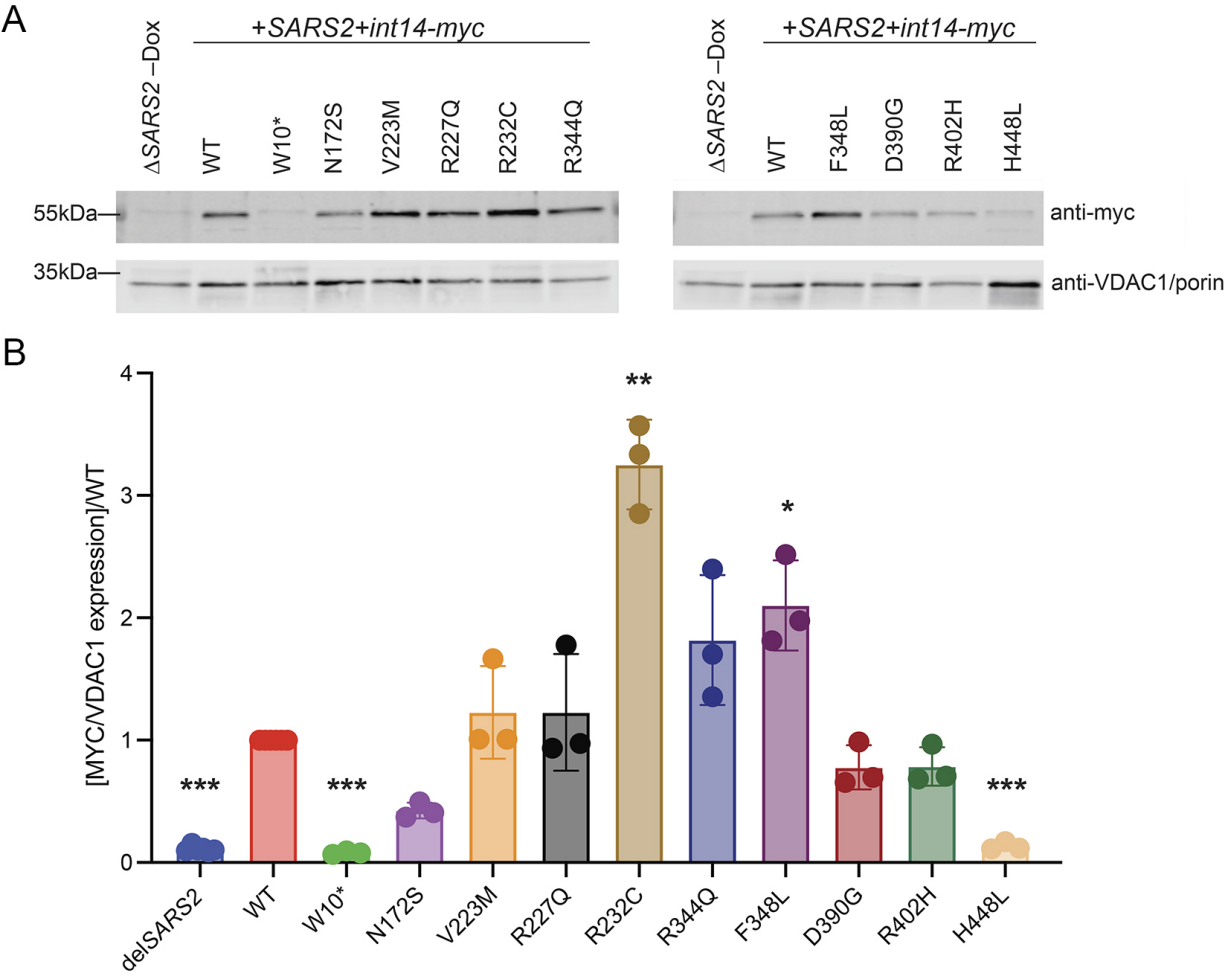
**Western blot analyses show variant-dependent effects on SARS2 protein levels.** (A) Representative western blot analysis performed on three independently isolated mitochondrial protein lysates. An anti-myc antibody was used to detect protein levels from the *SARS2+int14-myc* transgene. VDAC1 was used as a mitochondrial loading control. (B) Quantification of anti-myc expression normalized to VDAC1; wild-type SARS2+int14-myc was set to 1. Expression levels statistically different from WT as determined by paired two-tailed *t*-test are indicated (*P<0.05, **P<0.01 and ***P<0.001). *n*=3. Dox, doxycycline.

To study the effect of each variant on protein levels, Δ*SARS2+SARS2-1xFLAG* cells were transduced with either wild-type *SARS2+int14-myc* or the individual variants. After undergoing hygromycin selection, the transduced population of cells was grown without doxycycline for 6 days, and cells were harvested to isolate mitochondrial protein lysates. Because we (1) transfected cells with a multiplicity of infection (MOI) of 0.15 and (2) utilized transduced populations rather than isolating clonal lines, we ensured that cells would be transduced with only one copy of a virus and minimized negative impacts of site-specific integrations (e.g. on mRNA and protein expression), as every cell in each population would have different integration locations. We generated three sets of mitochondrial lysates per variant and performed western blot analyses using an antibody against the myc tag on the *SARS2+int14-myc* transgene on all of the variants except for c.1347G>A, which introduces a frameshift and would not express the myc tag (Fig. 5A; full-length blots for all replicates are provided in Fig. S6). We normalized the expression of each variant to VDAC1 as a mitochondrial loading control and then set the levels of wild-type SARS2+int14-myc protein to 1 on each western blot to account for differences in imaging exposure (Fig. 5B). We found that all variants except for W10* were expressed. H448L showed a statistically significant (*P*<0.001) decrease in protein levels compared to wild type, and R232C and F348L showed statistically significant (*P*<0.01 and *P*<0.05, respectively) increases in protein levels compared to wild type. N172S, D390G and R402H also appeared to show slight decreases in protein levels, although these findings were not statistically significant. To assess if reduced protein levels are associated with reduced transcript levels, we performed RNA-seq on three of the above samples (lentiviral transduced Hap1 cells grown in the absence of doxycycline for 6 days): wild type, R227Q (displaying protein levels similar to wild type) and H448L (displaying significantly reduced protein levels). Although R227Q and H448L transcripts were slightly lower than those of wild-type *SARS2*, there was no significant difference between R227Q and H448L *SARS2* (Fig. S7). In sum, these data suggest that a subset of pathogenic *SARS2* variants affect protein levels, while others do not impact protein levels and, in fact, may lead to an increase in protein levels; however, it is quite likely that this assay does not reflect protein levels in patient cells.

### Pathogenic *SARS2* variants have differential effects on mitochondrial oxygen consumption

We next employed our cell model to assess the impact of each pathogenic *SARS2* variant on mitochondrial function. Because SARS2 protein function is required for translation of the 13 mitochondrial-encoded oxidative phosphorylation proteins, we would expect that if a pathogenic variant reduces SARS2 protein function then this would result in decreased oxidative phosphorylation. Based on patient genotypes, we would expect that N172S, F348L, D390G, R402H, H448L and c.1347G>A would retain at least some function because they are found in the homozygous state in patients. Similarly, we would expect R232C to have some level of function because it is found *in trans* to W10* in the compound heterozygous state. Based on our protein expression data, we might also expect N172S, D390G, R402H and H448L to be associated with decreased oxygen consumption due to the decreased protein levels.

**Fig. 6.**
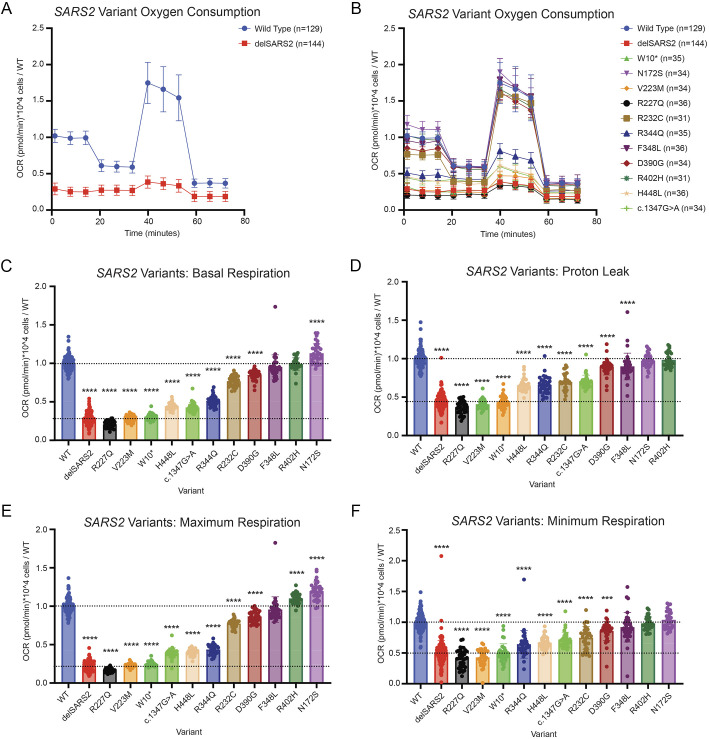
**Pathogenic *SARS2* variants differentially affect cellular oxygen consumption.** (A,B) Seahorse mitochondrial stress tests on Δ*SARS2+SARS2-1xFLAG* cells transduced with either wild-type *SARS2+int14-myc*, variant *SARS2+int14-myc* or no transduction. Oxygen consumption data are normalized by cell count. A shows exclusively the wild-type *SARS2+int14-myc* transduced cells and untransduced cells, and B shows all tested variants. (C-F) Oxygen consumption values for basal respiration (C), proton leak (D), maximal respiration (E) and minimum respiration (F) normalized to the average wild-type oxygen consumption on each plate. Dotted lines mark wild-type and untransduced oxygen consumption levels. One-way ANOVA tests were performed to assess statistical differences between all samples; *P*-values on bar graphs indicate values that are statistically different from wild-type *SARS2+int14-myc* oxygen consumption (***P<0.001; ****P<0.0001).

To test the effect of each pathogenic variant on mitochondrial oxygen consumption, we performed Seahorse mitochondrial stress tests on cell populations containing either (1) wild-type *SARS2+int14-myc*, (2) one of the 11 *SARS2+int14-myc* variants or (3) no transduced expression construct (Δ*SARS2+SARS2-1xFLAG* only cells, referred to as del*SARS2*). We grew each cell population without doxycycline for 6 days and then performed Seahorse assays. When we compared cells transduced with wild-type *SARS2+int14-myc* to untransduced cells, we observed a similar effect as when we tested Δ*SARS2+SARS2-1xFLAG* cells with versus without doxycycline, respectively (Fig. 6A), thus confirming the function of our wild-type expression construct. We used the above oxygen consumption rate (OCR) levels as a baseline for how wild-type *SARS2* or a complete null allele function in this assay and then compared these data to each pathogenic variant. When we overlaid the oxygen consumption results from each variant tested, we observed a range of effects on oxygen consumption (Fig. 6B; Fig. S8), demonstrating that certain pathogenic *SARS2* variants are more effective at complementing the loss of *SARS2-1xFLAG* expression than others.

To assess variant-specific oxygen consumption at each stage of the mitochondrial stress test, we normalized the oxygen consumption data of each variant to the average of wild-type *SARS2* oxygen consumption at the same stage: basal, proton leak, maximum and minimum (non-mitochondrial). We then arranged the allelic series at each stage in the order of function relative to that in wild-type *SARS2* (Fig. 6C-F). At all stages, we observed variant-specific differences in oxygen consumption compared to wild-type *SARS2*. We observed that certain variants exhibited a complete loss-of-function effect similar to the *SARS2* knockout (e.g. R227Q at all stages), that certain variants showed hypomorphic effects (e.g. R344Q, which has 32% basal respiration relative to wild type and 26% maximal respiration relative to wild type), and that certain variants behaved similarly to wild-type *SARS2* (e.g. R402H, which has 98% basal respiration relative to wild type and 112% maximal respiration relative to wild type). This demonstrated that we can use mitochondrial respiration as a tool to identify functional differences between pathogenic variants.

### D390G and R402H *SARS2* impact post-transcriptional processing

The R402H missense variant is the most commonly reported pathogenic *SARS2* variant thus far (Colin et al., 2021; Rivera et al., 2013; Roux et al., 2021; Yang et al., 2022; Zhou et al., 2021). It was, therefore, unexpected that Seahorse assays revealed no differences in oxygen consumption between R402H and wild-type *SARS2* (Fig. 6). A major limitation of our model system is that *SARS2* alleles are expressed via a cDNA construct (with the addition of intron 14). We therefore hypothesized that R402H *SARS2* may impact post-transcriptional processing (e.g. splicing) that would not be detectable in this model. Assessment of this variant in the Human Splicing Finder Mutation Analysis Tool (Desmet et al., 2009) revealed that R402H is predicted to modify the exonic splice enhancer/exonic splice silencer ratio by ablating three enhancers and introducing one silencer (exonic splicing enhancer/exonic splicing silencer motifs ratio −4), potentially negatively impacting the rate of exon 13 inclusion into *SARS2* transcripts.

**Fig. 7.**
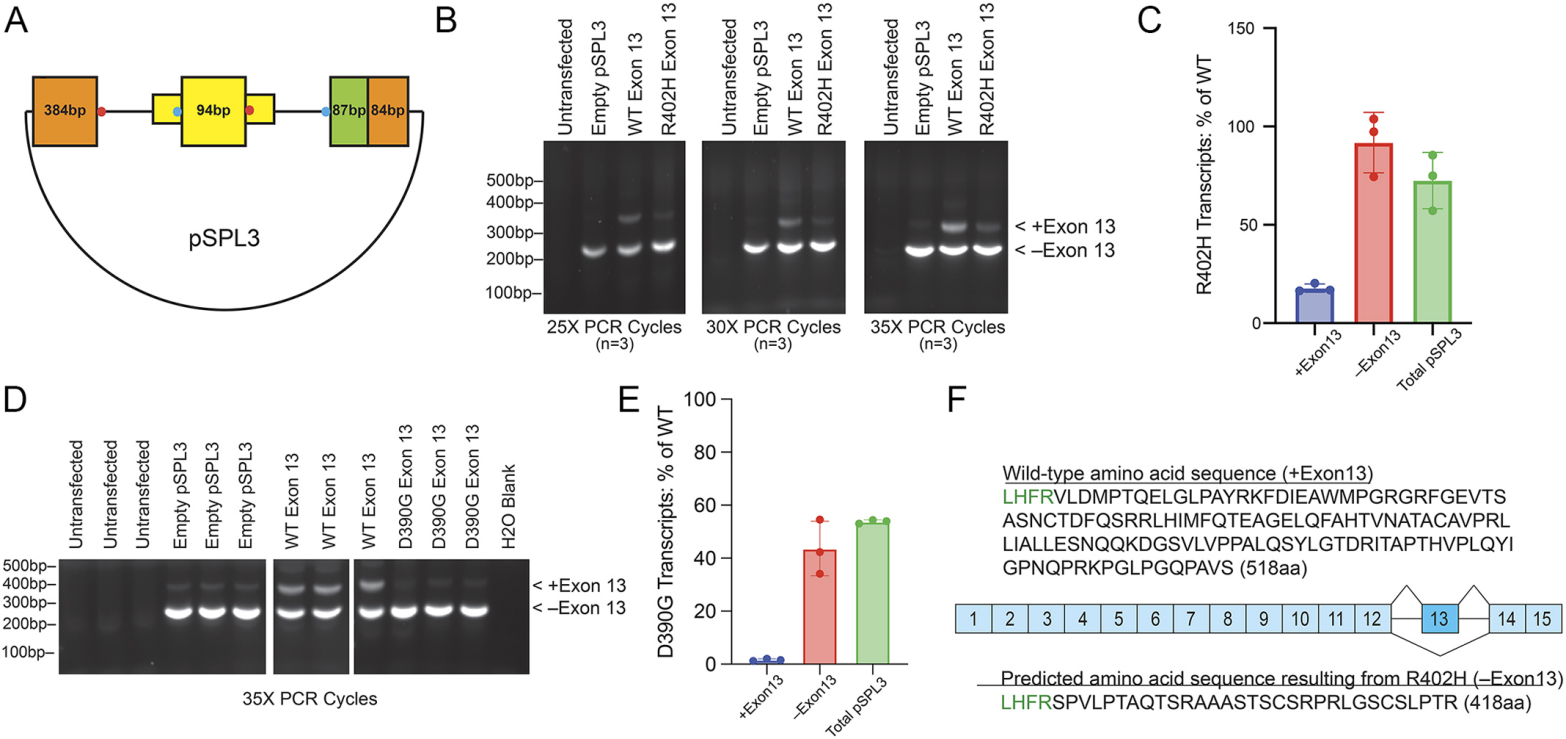
**D390G and R402H *SARS2* impact inclusion of exon 13 in minigene assays.** (A) Minigene plasmid design to assess *SARS2* exon 13 splicing. Large orange boxes indicate exons derived from the rabbit beta-globin gene; large green box indicates synthetic exon sequence; large yellow box indicates *SARS2* exon 13; and smaller yellow boxes indicate endogenous *SARS2* upstream (102 bp of intron 12) and downstream (91 bp of intron 13) intronic sequences. Exon lengths are indicated in each box. Black lines between exons correspond to synthetic intron sequences. Red and blue dots correspond to splice donor and acceptor sites, respectively. (B) Representative gels showing PCR analysis to assess inclusion or exclusion of wild-type or R402H *SARS2* exon 13. Products with exon 13 included are 351 bp, and products with exon 13 excluded are 257 bp. Samples are indicated across the top, and a base pair ladder is shown on the left. Gels shown contain PCR reactions run for 25 (left), 30 (middle) and 35 (right) cycles. *n*=3. (C) Quantification of alternatively spliced minigene products by qPCR analysis of three independent RNA isolates. Each transcript of interest is normalized to the housekeeping gene *REEP5.* Fold changes were calculated by taking 2^−ΔΔCt^ and multiplied by 100 to obtain a percentage of each transcript in samples transfected with R402H exon 13 compared to samples transfected with wild-type exon 13. (D) Representative gels showing PCR analysis to assess inclusion or exclusion of wild-type or D390G *SARS2* exon 13, run under the same conditions as shown in B. Three independent RNA samples were assessed for each sample, and reactions were run for 35 cycles. *n*=3. (E) Quantification of alternatively spliced minigene products by qPCR analysis of three independent RNA isolates. Fold changes were calculated as described in C to compare samples transfected with D390G exon 13 to samples transfected with wild-type exon 13. (F) Model of predicted consequences of exon 13 exclusion in *SARS2* transcripts. Green amino acids indicate unchanged residues, and black amino acids correspond to those following exon inclusion or exclusion.

To interrogate R402H *SARS2* for an effect on gene splicing, we developed a minigene assay to assess the rate of exon 13 inclusion by inserting the entire *SARS2* exon 13 (94 bp, wild type or R402H) and ∼100 bp of flanking intronic sequences into the pSPL3 minigene plasmid (Fig. 7A) (Tompson and Young, 2017). We included 102 bp of intron 12 and the entirety of intron 13 (91 bp). Resulting plasmids were transfected into HEK-293T cells, and cell populations were harvested for RNA isolation, cDNA generation, and PCR amplification of the minigene transcript product. DNA gel electrophoresis indicated a decreased band intensity for the PCR product containing R402H exon 13 compared to the wild-type exon 13 (Fig. 7B). To complement these studies, we designed a quantitative reverse transcription PCR (qRT-PCR) assay using primers specific to either exon 13 or to the synthetic exon–exon boundary, targeting exon-skipped products. These assays revealed that cells transfected with the R402H minigene express 17.8% of the exon 13-containing transcript compared to cells transfected with the wild-type minigene plasmid (Fig. 7C). These findings suggest that the R402H variant decreases the rate of exon 13 inclusion into the *SARS2* transcript, which would cause a frameshift and would likely subject the transcript to nonsense-mediated decay (Fig. 7F). Interestingly, when we assessed the total minigene transcript using primers specific to the downstream synthetic exon, we also observed a general decrease in pSPL3 transcripts in cells transfected with the R402H minigene compared to the wild-type minigene: cells transfected with the R402H minigene had ∼75% of the wild-type level of total pSPL3 transcripts, suggesting that the R402H variant may also affect transcript stability, although this would need to be tested directly (Fig. 7C).

Given these findings, we also assessed the D390G *SARS2* variant, which had modest effects on mitochondrial function in our Seahorse assay, leads to a severe clinical phenotype and also maps to *SARS2* exon 13. We therefore generated an additional pSPL3 vector containing the D390G variant in exon 13 and repeated our splicing PCR and qPCR experiments on cDNA and RNA isolated from transfected HEK-293T cells. DNA gel electrophoresis also indicated a decreased band intensity for the PCR product containing D390G exon 13 compared to wild-type exon 13, and qRT-PCR assays found that cells transfected with the D390G minigene express 1.6% of the exon 13-containing transcript compared to cells transfected with the wild-type minigene plasmid (Fig. 7D,E). Similar to our experiments on R402H, the total pSPL3 transcript was also decreased in cells transfected with the D390G exon 13 minigene compared to wild type (54%), suggesting the D390G variant may also lead to transcript instability in addition to impacting splicing patterns. In sum, our data support the hypothesis that the post-transcriptional effects of D390G and R402H *SARS2*, rather than protein function effects, contribute to the impaired gene function in patients carrying this allele.

### Genotype–phenotype assessments

As noted, pathogenic *SARS2* variants cause two distinct recessive phenotypes, HUPRA syndrome and spastic paresis (SP). To consider the functional consequences of each allele in the context of the patient genotype, we added the maximum oxygen consumption values of each allele in each genotype, taking into account the effect that D390G and R402H *SARS2* have on exon 13 inclusion. We then compared these data to the wild type/wild-type genotype (set to 100% function, with delSARS2 set to 0% function). This revealed that five genotypes associated with HUPRA syndrome result in more severe gene depletion than the two genotypes associated with SP (Table 2), consistent with the more severe phenotype of HUPRA syndrome. Interestingly, all five of the above genotypes contain D390G or R402H. In contrast, three genotypes associated with HUPRA syndrome showed less severe gene depletion than the SP-associated genotypes (Table 2); these three genotypes are composed of three variants – N172S, F348L and R232C. There are at least two possible explanations for this surprising finding. First, the missense variants in the less functionally impactful genotypes may not be pathogenic; indeed, two variants (R232C and F348L) were collected from ClinVar, and the genetic argument supporting pathogenicity is not clear. Second, the missense variants in the less functionally impactful genotypes may impair gene function in ways not tested in our assays, similarly to D390G and R402H. Further studies are, therefore, needed on these three variants, including genetic studies to more carefully assess pathogenicity, and *in vitro* enzyme kinetic and protein and RNA expression studies on patients samples to more broadly evaluate the impact of these variants on gene function. Such analyses will be essential for improving our understanding of genotype–phenotype correlations in patients with *SARS2*-related disease.

**Table 2.** Summary of effects of *SARS2*-associated variants by genotype on oxygen consumption by genotype adjusted for splicing deficiencies

Genotype	Allele 1	Allele 2	Percentage maximal respiration versus that of WT	Phenotype
WT/WT	50%	50%	100%	N/A
N172S/N172S	63%	63%	126%	HUPRA syndrome
F348L/F348L	47%	47%	94%	HUPRA syndrome
W10*/R232C	2%	36%	38%	HUPRA syndrome
H448L/H448L	12%	12%	24%	Spastic paresis
c.1347G>A/c.1347G>A	11%	11%	22%	Spastic paresis
R344Q/R402H	13%	8.5%	21.5%	HUPRA syndrome
R402H/R402H	8.9%	8.9%	17.8%	HUPRA syndrome
V223M/R402H	1%	8.9%	9.9%	HUPRA syndrome
R227Q/R402H	0%	8.9%	8.9%	HUPRA syndrome
D390G/D390G	0.8%	0.8%	1.6%	HUPRA syndrome

N/A, not applicable; WT, wild type.

## DISCUSSION

This study characterizes the role of *SARS2* in the cell by assessing the transcriptional changes that result from depleting *SARS2* expression in Hap1 cells and identifies variant-dependent effects on mitochondrial oxygen consumption. We show that the absence of *SARS2* expression potentially activates cellular stress responses, likely reacting to defects in mitochondrial translation. We also show that pathogenic variants cause a range of distinct changes to mitochondrial oxygen consumption levels. Finally, we demonstrate that, although many *SARS2* variants cause loss-of-function effects due to defects in protein function, some missense changes, such as D390G and R402H, may cause post-transcriptional defects that are responsible for decreased gene function.

In the process of assessing the transcriptional changes that result from the loss of *SARS2* expression in our Δ*SARS2+SARS2-1xFLAG* cells, our findings validate expectations that the loss of *SARS2* will lead to mitochondrial dysfunction and indicate that cellular stress pathways are activated. These pathways include the HRI kinase branch of the ISR, the UPR and ER stress. Identification of the HRI branch of the ISR in our REACTOME analyses is consistent with evidence supporting HRI as the kinase that activates the ISR during mitochondrial stress (Chakrabarty et al., 2024; Guo et al., 2020; Wu et al., 2024). In addition, the UPR and ER stress are known to be activated in response to mitochondrial stress (Chu et al., 2019; Zhu et al., 2021). There have been numerous studies in recent years indicating that the ISR is activated in both cytoplasmic and mitochondrial ARS-mediated disease (Agnew et al., 2018; Dogan et al., 2014; Jin et al., 2021; Spaulding et al., 2021).

This study also provides key insight into the pathological mechanisms of *SARS2*-mediated disease. Interestingly, our oxygen consumption and splicing data, combined, indicate that patients with HUPRA syndrome may have less *SARS2* gene function than patients with PSP (five genotypes, Table 2). This supports expectations given our current understanding of patient lifespan, which tends to be shorter in patients with HUPRA syndrome than in patients with PSP. However, three genotypes associated with HUPRA syndrome are predicted to function better than the two PSP-associated genotypes (Table 2). As noted above in the ‘Genotype–phenotype assessments’ section, additional studies are required to test the pathogenicity and functional consequences of the three missense variants identified in these genotypes.

It is important to point out that the Seahorse assay leveraged in this study has a number of limitations that might be contributing to incomplete assessment of the effect of each *SARS2* variant. First, the Δ*SARS2+SARS2-1xFLAG* cells were generated using Hap1 cells, which were derived from leukemia cells (Llargues-Sistac et al., 2023). With the exception of the hyperuricemia and alkalosis phenotypes, Hap1 cells are largely not a relevant cell type for studying HUPRA syndrome or PSP; *SARS2* variants might have different effects in other cell types. Our protein expression data indicate that a subset of variants lead to decreased SARS2 protein levels, while other variants show an increase in protein levels; however, as these data were gathered in Hap1 cells containing *SARS2* transgenes expressed via a strong promoter, assessing protein levels in a panel of patient cells or in a more disease-relevant cell type might lead to different results. Indeed, this may affect our interpretation of the oxygen consumption data, as some variants that showed little impact on oxygen consumption appear to demonstrate lower protein levels than wild type (e.g. N172S), suggesting that, although a decrease in oxygen consumption was not detectable in our assay, it could be observed in a more relevant cell type (Table 2).

As noted above, another limitation of our model is that each variant is tested in the context of the open reading frame, with the exception of the inclusion of intron 14. It is possible that some variants – especially those that behave similarly to wild type and do not show any defects in mitochondrial function – may affect transcriptional processing that cannot be detected in our model. This is evidenced by our studies on D390G and R402H, which indicate a potential decrease in exon inclusion. Skipping exon 13 would lead to a frameshifted transcript that may be subject to nonsense-mediated decay. Importantly, when we adjust our expected maximal respiration values to account for decreased exon inclusion, genotypes containing the D390G and R402H variants appear to be the most severe (Table 2). Although there are limitations to this approach given that these effects were observed using cells transfected with minigene plasmids rather than by assessing splicing modifications in the full genomic context, it emphasizes the importance of assessing post-transcriptional impacts of variants that may otherwise have minimal effects in functional assays. Ideally, the effect of D390G and R402H on *SARS2* splicing should be confirmed in patient samples; however, such samples are currently unavailable.

It is also important to consider other potential factors that might be causing disease aside from oxygen consumption. As evidenced by our RNA-seq findings, multiple cellular stress responses may be active in cells with depleted *SARS2* function. It is possible that the downstream consequences of stress response activation may be contributing to disease phenotypes, even if the direct impact of variants on protein function may not be as severe. Studies on glycyl-tRNA synthetase variants (which functions in the cytoplasm and the mitochondria) show that inactivating the ISR rescues disease phenotypes despite not directly resolving the translation defects (Spaulding et al., 2021). Other studies on mitochondrial disease have also shown that tempering stress responses rescues phenotypes without directly resolving the consequence of the genetic defect (Han et al., 2023). In the context of *SARS2*-related recessive disease, the above observations raise two non-mutually-exclusive hypotheses: (1) activation of stress pathways is beneficial, in the short term, for cell survival upon *SARS2* depletion; and (2) chronic, long-term activation of stress pathways exacerbates the patient phenotype. Although future work is needed to confirm that dysfunctional *SARS2* directly leads to cellular stress response activation and to test the two above-mentioned hypotheses, addressing these questions will be important for determining whether inhibiting stress responses is a viable therapeutic for patients with *SARS2*-related disease. Alternatively, studies have revealed noncanonical functions for cytoplasmic and mitochondrial ARSs, including roles in angiogenesis and mTORC1 activation (Kim et al., 2021; Li et al., 2021; Wang et al., 2016). It is possible that some variants impact unappreciated noncanonical functions of the SARS2 protein that would not be detected in our assays.

Finally, additional research is needed to define the clinical spectrum of *SARS2*-related disease. Although patients largely fall into the categories of HUPRA syndrome or PSP, many patients present with additional phenotypes that are unique to particular genotypes. For example, the patient reported with the genotype R344Q/R402H presents with HUPRA syndrome phenotypes (renal failure, pulmonary hypertension) and non-HUPRA phenotypes (sideroblastic anemia) (Colin et al., 2021). Similarly, a patient with the genotype c.654-14T>A/c.1519dupC (variants not tested in this study owing to our inability to model the intronic variant) was reported to have HUPRA syndrome phenotypes and PSP phenotypes, including epilepsy, cerebellar atrophy, increased muscle tone and paralysis (Yu et al., 2022). These observations indicate that *SARS2*-related disease does not consist of binary phenotypic outcomes but likely represents a spectrum of clinical phenotypes. Precise and thorough patient phenotyping is now required to better define how variant-specific functional data correlate with patient genotypes and phenotypes.

Overall, the studies described here broaden our understanding of the impact of depleted *SARS2* expression in cells, demonstrate preliminary associations between oxygen consumption levels and clinical phenotypes, and provide novel insights into the mechanisms by which *SARS2* variants cause disease.

## MATERIALS AND METHODS

### Lentiviral vector generation

The *SARS2* open reading frame was amplified from cDNA (generated from RNA isolated from control fibroblast cells) using Phusion DNA Polymerase (Thermo Fisher Scientific) and primers designed at the *SARS2* start and stop codons. The resulting PCR product was gel extracted and cloned into the pDONR 221 vector using Gateway BP cloning (Invitrogen), which was transformed into *Escherichia coli* and grown on LB medium containing kanamycin. Individual colonies were selected and subjected to DNA isolation using an QIAprep Miniprep Kit (Qiagen). DNA was digested with BsrGI-HF to confirm successful recombination, and correctly digesting samples were subjected to sequencing analysis to rule out any polymerase-induced errors. Next, coding sequences for a 1×FLAG C-terminal tag and PamX mutations to prevent cutting by Cas9 in subsequent experiments (see below) were introduced using the QuickChange II Site-Directed Mutagenesis kit (Agilent) and mutagenic primers (Table S1). Mutagenesis reactions were transformed into *E. coli* and grown on LB medium containing kanamycin. After subjecting transformants to sequencing to confirm the introduction of each desired mutation and rule out any other polymerase-induced errors, the resulting modified *SARS2* open reading frame was then cloned into the pCW57.1 lentiviral vector (Addgene plasmid #41393) using Gateway LR cloning (Invitrogen). Individual clones were selected and grown on LB medium containing ampicillin. DNA from each clone was isolated using a Plasmid Plus Midi kit (Qiagen) before submitting to the University of Michigan Vector Core for lentiviral production.

Experimental expression constructs containing the *SARS2* open reading frame in addition to intron 14 (at the naturally occurring position between exon 14 and 15 coding sequences) and a C-terminal myc tag (*SARS2+int14-myc*) that are either wild type or that contain the R402H or the c.1347G>A splice-site variant were synthesized and sequence verified (Twist Biosciences). Wild-type *SARS2+int14-myc* was mutagenized to each of the additional variants studied using a QuickChange II Site-Directed Mutagenesis kit (Agilent) along with variant-specific primers (sequences available upon request). Mutagenic reactions were transformed into *E. coli* and grown on LB medium containing kanamycin. DNA was subsequently isolated from individual colonies, and the DNA was subjected to sequence analysis to confirm the presence of the desired variant and the absence of any polymerase-induced errors. Resulting constructs were cloned into the pLENTI PGK Hygro DEST (w530-1) vector (Addgene) using Gateway LR Cloning (Invitrogen), transformed into *E. coli* and grown on LB medium containing ampicilin. Individual colonies were selected and grown in liquid media, and DNA was isolated using the Plasmid Plus Midi kit (Qiagen) before submitting to the University of Michigan Vector Core for lentiviral production.

### sgRNA design and cloning

sgRNAs within exons 11 and 14 of the endogenous *SARS2* gene were selected using the UCSC Genome Browser CRISPR/Cas9 NGG Targets track. Single-stranded sgRNA oligonucleotides were synthesized (IDT) and then phosphorylated and annealed in 10 μl reactions [using 1 μl of each 100 μM oligonucleotide, 1 μl 10× T4 ligase with ATP, 0.5 μl T4 PNK (NEB) and 6.5 μl H_2_O] annealing at 37°C for 30 min, 95°C for 5 min and then ramping down to 25°C by 5°C/min. The resulting reaction was diluted 1:250 and then cloned into either the pSpCas9(BB)-2A-Puro (px459, Addgene plasmid #62988) V2.0 backbone or the pSpCas9(BB)-2A-GFP (px458, Addgene plasmid #48138) backbone using Fast Digest *Bpi*I (Thermo Fisher Scientific) and T7 DNA Ligase (NEB) with 1 mM DTT and 1 mM ATP. The reaction was rotated between incubations at 37°C for 5 min and 23°C for 5 min a total of six times.

Optimal sgRNAs were identified by transfecting HEK-293T cells with individual sgRNAs in the px459 backbone and assessing the amount of insertions and deletions introduced, as a proxy for cutting by Cas9. Transfected cells were selected for using 2.5 μg/ml puromycin for 2 days, and the resulting cell population was harvested for gDNA isolation using a Wizard Genomic DNA Purification kit (Promega). gDNA underwent Tn5 tagmentation library preparation, which fragments and tags gDNA with adaptors simultaneously. DNA samples were diluted to 10 ng/μl, and 1 μl was added to 4 μl 5× TagBuffer, 1 μl Tn5 enzyme with adaptors and 14 μl H_2_O (provided by Dr Jacob Kitzman, University of Michigan, Ann Arbor, MI, USA). Samples were incubated at 55°C (lid 65°C) for 7 min, then supplemented with 5 μl 0.2% SDS and incubated for another 15 min under the same conditions. Resulting reactions were PCR amplified to attach barcoded primers (provided by Dr Jacob Kitzman) using Kapa Hifi Non-Hotstart Polymerase (Roche). Reactions contained 5 μl 5× Kapa Fidelity Buffer (Roche), 0.75 μl 10 mM dNTPs, 0.63 μl 10× SYBR Green I (Thermo Fisher Scientific), 0.5 μl 10 μM forward and reverse inner primers, 1 μl 0.25 μM forward and reverse barcode primers, 0.5 μl Kapa Hifi Polymerase (Roche), 5 μl template DNA and 10.12 μl dH_2_O. Reactions underwent qRT-PCR and were amplified until plateau, and resulting PCR products were column purified and then submitted for sequencing using the Illumina MiSeq. The percentage of reads containing insertions or deletions at the sgRNA site was calculated for each sgRNA; sgRNAs with an indel rate of over 20% were considered ‘efficient’ and selected for future use (sgRNA 1, GGTGCTGGAGCAAACCATCC; sgRNA 2, GTCTGTGCAGTTGGAAGCAC).

### Lentivirus generation and lentiviral transductions

Lentiviruses were made by the University of Michigan Vector Core. Briefly, HEK-293T cells were transfected with proviral plasmid, Gag-pol viral capsid plasmid (psPAX2) and VSVG envelope plasmid (pMD2.G). 48-72 h post transfection, medium was harvested and spun to remove cellular debris or passed through a low-protein binding 0.45 μm filter. Viral supernatant was transferred to a new tube and stored at −80°C. The University of Michigan Vector Core produces viral lysates with ∼1E+6 TU/ml (1× concentration, based on a control virus). Viruses were diluted to a MOI of 0.15, which was confirmed by counting cells that survived after selection of drug resistant cells transduced with increasing amounts of virus.

1.2 million Hap1 cells were plated and simultaneously transduced with lentiviruses at a 0.15 MOI and grown in Iscove's modified Dulbecco's medium (IMDM; Gibco) at 37°C with 5% CO_2_ (standard mammalian cell culture conditions) overnight. The next day, cells were treated with the appropriate antibiotic (2.5 μg/ml puromycin for pCW57.1 lentiviruses; 1 mg/ml hygromycin for pLenti PGK Hygro lentiviruses) to select for transduced cells. Cells treated with puromycin were grown for 2 days under selection before recovery, and cells treated with hygromycin were grown for 6 days under selection before replacing medium with fresh IMDM without puromycin or hygromycin to recover from selection under standard mammalian cell culture conditions for 1-3 days depending on confluency of the flask.

### CRISPR transfections and cell line expansion

Transfections were performed using Lipofectamine 3000 (Thermo Fisher Scientific). For transfections performed in six-well plates (500,000-1 million cells), 500 ng of each transfected plasmid was added to each sample. For *SARS2* knockout transfections in T75 flasks, plates of transduced cells that survived drug selection were treated with 8 μg of each sgRNA. 2 days after transfection, cells were harvested and sorted for GFP-positive expression in the University of Michigan Flow Core using an MA900 Cell Sorter (Sony); GFP gating was performed using untransduced and untransfected control Hap1 cells and untransduced Hap1 cells transfected with one sgRNA. Single GFP-positive cells were plated into 96-well plates in IMDM containing 20 ng/ml doxycycline and grown at standard mammalian cell culture conditions, and surviving colonies were expanded into clonal lines. PCR genotyping around the expected deletion was performed to validate the presence of the deletion. Primers were designed upstream of exon 10 (in intronic sequence to avoid amplifying *SARS2-1xFLAG*) and within exon 15; PCR amplification would capture the expected deletion region between exons 11 and 14. Cell lines were selected which contained a PCR product that reflected the smaller band – indicating a deletion product, and no larger band – indicating an unedited product. Selected cell lines are maintained with continuous treatment of 15 ng/ml doxycycline.

### Whole-cell and mitochondrial protein isolation

Frozen cells harvested from Δ*SARS2+SARS2-1xFLAG* experiments were resuspended in 50 μl (from six-well plates) or 100 μl (from 10 cm dishes) of RIPA buffer with 100× Proteinase Inhibitor Cocktail (Thermo Fisher Scientific) and incubated on a rocker at 4°C for 30 min. Next, the samples were centrifuged at 13,000 ***g*** for 30 min to collect cellular debris. The resulting protein-bearing supernatants were carefully removed from the precipitate and aliquoted into fresh 1.5 ml tubes. Protein concentrations were quantified via Pierce BCA (Thermo Fisher Scientific) on a BioMate3 (Thermo Fisher Scientific) to measure the 562 nm intensity that corresponds with increasing protein concentrations. Lysates were stored at −80°C.

To isolate mitochondrial protein, mitochondria were isolated from *SARS2+int14-myc* experiments using a Mitochondrial Isolation Kit for Cultured Cells (Thermo Fisher Scientific); Protocol Option A (using Reagent B rather than sonication) was followed. Resulting mitochondrial pellets were processed for protein isolation as described above and using 40 μl RIPA buffer and 100× Proteinase Inhibitor Cocktail.

### Western blot analysis

Protein samples were prepared with 25 μg whole-cell protein lysate or 2.5 μg mitochondrial protein lysate. Protein was added to 12.5 μl of Novex Tris-Glycine SDS 2× sample buffer (Thermo Fisher Scientific), and the volume was brought up to 25 μl with H_2_O before adding 1 μl β-mercaptoethanol. Samples were boiled at 99°C for 5 min and then loaded onto a 4-20% Tris-glycine gel (Invitrogen) along with 2 μl PageRuler Plus Ladder (Thermo Fisher Scientific). Gels were run in the Thermo Fisher Scientific Mini Gel Tank for 75 min at 150 V. Membranes were activated using methanol and then incubated in transfer buffer (860 ml water+40 ml Novex 25× Tris-glycine transfer buffer concentrate+100 ml methanol). Transfers were run for 1 h at 100 V in a Bio-Rad Mini Trans-Blot Cell. Post-transfer membranes were blocked with 5% milk in PBS or Tris-buffered saline with Tween-20 (TBST) for 1 h and then incubated with primary antibody in 5% milk overnight at 4°C. Primary antibodies used include anti-SARS2 (1:1000; Thermo Fisher Scientific PA5-84321); anti-IARS1 (1:10,000; GeneTex GTX131733); anti-FLAG (1:2500; BioLegend 637302); anti-myc (1:500; Thermo Fisher Scientific PA1-981); anti-VDAC1 (1 μg/ml; Abcam ab15895). The next day, membranes were washed with TBST and then incubated with LiCor green anti-rabbit secondary antibodies (1:10,000; IRDye 800CW goat anti-rabbit, LiCor 926-32211) for 1 h. Samples were washed again with TBST and then imaged using a LiCor CLx.

### Seahorse mitochondrial stress tests

For Seahorse assays, cells were plated at a density of 80,000 cells/well in Seahorse 96-well plates (Agilent) in standard IMDM (80 μl/well). Cells sat at room temperature (RT) for 1 h before being incubated overnight in standard mammalian cell culture conditions (see above). The next day, the IMDM was replaced with Seahorse XF DMEM supplemented with 25 mM glucose, 4 mM glutamine and 1 mM pyruvate (Agilent Seahorse XF DMEM assay medium pack, pH 7.4). IMDM was removed and replaced with 180 μl Seahorse DMEM, and then 150 μl Seahorse DMEM was removed and replaced twice to wash remaining IMDM from wells. Cells were incubated in a non-CO2 37°C incubator for 1.5 h to allow cells to adjust to new media conditions. Immediately before the experiment, 60 μl Seahorse DMEM was removed and replaced from each well. Oxygen consumption data were collected using the Seahorse XFe96. During the experiment, three baseline OCR measurements were made before cells were treated with 1 μM oligomycin, 1 μM FCCP and 0.5 μM Antimycin A/Rotenone (Agilent). Under each condition, the plate was mixed for 3 min before being read for 3 min (for a total of 3 readings over the course of 18 min per condition). After the experiment concluded, total cells per well were counted using brightfield microscopy on a Cytation 5 (BioTek), and raw OCR readings for each well were normalized to the cell counts for that same well.

### RNA isolation

RNA was isolated from Δ*SARS2+SARS2-1xFLAG* cells grown with doxycycline or without doxycycline for 3, 6 or 9 days using Trizol/chloroform and an RNeasy Mini Kit (Qiagen). Frozen cell pellets were resuspended in Trizol, and then chloroform was added at 1/5 volume of Trizol. Samples were vigorously vortexed for 30 s, incubated at RT for 1 min, and then centrifuged in a microcentrifuge at 13,000 ***g*** for 15 min at 4°C. After centrifugation, the aqueous layer of each sample was transferred to a new 1.5 ml tube with an equal volume of 70% ethanol. Each resulting sample was added to an RNeasy column (Qiagen) and then processed through the remainder of the RNeasy Mini Kit, starting with centrifugation and washing with Buffer RW. First, RNA samples were centrifuged at 13,000 ***g*** for 1 min. Flow-through was discarded, and then column was washed with 350 μl Buffer RW. After spinning at 13,000 ***g*** and discarding flow-through, 80 μl DNase I working mixture (10 μl DNase I+70 μl of buffer) (Qiagen RNase-free DNase set) was added to each column and incubated at RT for 15 min. After incubation, 350 μl Buffer RW was added to each column, followed by centrifugation at 13,000 ***g*** for 1 min. Columns were washed with 500 μl Buffer RPE and centrifuged twice; then, after discarding the flow-through, spun for a ‘dry spin’ at 13,000 ***g*** for 1.5 min. 35-50 μl H_2_O was added to each column and incubated at RT for 5 min, then RNA was eluted by spinning at 11,000 ***g*** for 1 min. Samples were stored at −80°C.

### RNA-seq

RNA isolated from Δ*SARS2+SARS2-1xFLAG* cells grown with doxycycline or without doxycycline for 3, 6 or 9 days was submitted to the University of Michigan Advanced Genomics Core (AGC) for RNA sequencing using Illumina sequencing. The AGC generated a polyA-enriched RNA sequencing library and performed sequencing on the NovaSeq S4. Trimmed reads were aligned to the GRCh38 reference genome using HISAT2 (Kim et al., 2019) and then sorted using samtools (Danecek et al., 2021). Resulting bam files were analyzed using DESeq2 (Love et al., 2014) to assess differential gene expression. Volcano plots were generated using gglplot (Wickham, 2016). Venn diagrams were generated using an online tool. GO term and REACTOME analyses were performed using the Gene Ontology Resource and the REACTOME Pathway Browser (Ashburner et al., 2000; Fabregat et al., 2017; Gene Ontology Consortium et al., 2023). Heat maps were generated using Pheatmap.

To correlate differences in SARS2 protein levels with differences in *SARS2* transcript levels, RNA was isolated (as above) from lentiviral transduced Hap1 cells grown in the absence of doxycycline for 6 days and expressing wild-type, R227Q or H448L *SARS2*. These RNA samples were subjected to commercially available RNA-seq analysis (Plasmidsaurus).

### qPCR

RNA isolated from Δ*SARS2+SARS2-1xFLAG* cells grown with doxycycline or without doxycycline for 3, 6 or 9 days was converted into cDNA using an Applied Biosystems High-Capacity cDNA Reverse Transcription Kit with RNAse Inhibitor (Thermo Fisher Scientific). Reactions were set up according to the kit manual [e.g. one 20 μl reaction included 2 μl 10× RT Buffer, 0.8 μl 25× dNTP mix, 2 μl 10× Random Primers, 1 μl Reverse Transcriptase, 1 μl RNAse Inhibitor, 5 μl 2 ng/μl RNA (1 μg) and 8.2 μl H_2_O]. Resulting cDNA samples were diluted to 20 ng/μl for qPCR. qPCR reactions were set up using TaqMan Gene Expression Master Mix (Thermo Fisher Scientific) and gene-specific TaqMan Gene Expression Assays with FAM fluorescence (Thermo Fisher Scientific); 100 ng cDNA was added to each well except for ‘no template’ controls, which instead included 5 μl of sterile, molecular biology-grade water. Specific gene expression assays used included *GDF15* (Hs00171132_m1), *DDIT3* (Hs00358796_g1), *CHAC1* (Hs00225520_m1), *TRIB3* (Hs01082394_m1) and *ATF5* (Hs01119208_m1). qPCR was performed on an Eppendorf MasterCycler realplex2, and ΔΔCt values were calculated using *REEP5* (Hs00382478_m1) expression as the control.

### Multiple species alignment

The Clustal Omega program was used to align SARS2 protein sequences from multiple species. The following GenBank accession numbers were used to generate the alignment: human (*Homo sapiens*, NP_060297.1), cow (*Bos taurus*, NP_776882.1), mouse (*Mus musculus*, NP_076126.2), rat (*Rattus norvegicus*, NP_001099710.1), zebrafish (*Danio rerio*, NP_957473.1), fly (NP_001262599.1), worm (*Caenorhabditis elegans*, NP_500549.1) and yeast (*Saccharomyces cerevisiae*, NP_011875.1).

### Splicing minigene restriction enzyme cloning

The pSPL3 was provided by Dr Jacob Kitzman, and the wild-type and mutant 287-bp genomic regions surrounding human *SARS2* exon 13 (102 bp of intron 12, the full 94 bp of exon 13 and the full 91 bp of intron 13) were commercially synthesized and sequence verified (Twist Bioscience) with flanking *Pst*I and *Xho*I restriction enzyme recognition sequences to allow directional cloning into pSPL3. The pSPL3 plasmid was digested using *Pst*I and *Xho*I (NEB) at 37°C for 3 h, and the linearized backbone was dephosphorylated using Antarctic Phosphatase (NEB) at 37°C for 3 h. The backbone was purified using a DNA Clean and Concentrator Kit (Zymo Research). In parallel, insert-containing entry vectors were digested using *Pst*I and *Xho*I at 37°C for 3 h, and the insert was purified via gel extraction. Each insert was ligated into pSPL3 using Quick Ligase (NEB) at a 7:1 molar ratio of insert:vector for 15 min at RT before being transformed into *E. coli* and selecting on LB medium containing ampicillin. Individual colonies were selected, and isolated DNA was verified via full-plasmid sequencing.

### Alternative transcript quantification

HEK-293T cells were transfected with pSPL3 plasmids using Lipofectamine 3000 (Thermo Fisher Scientific). 500,000 cells were seeded into six-well plates the day before transfection, and then cells were transfected with 500 ng plasmid DNA. Transfected cells were grown for 2 days before harvesting. RNA was isolated from resulting cells. For visualization of transcript products, RNA was converted into cDNA using the Applied Biosystems High-Capacity cDNA Reverse Transcription Kit with RNAse Inhibitor (Thermo Fisher Scientific). Reactions were set up according to the kit manual. PCR was performed using 2 μl resulting cDNA and pSPL3-specific primers (F, 5′-TCTGAGTCACCTGGACAACC-3′; R, 5′-ATCTCAGTGGTATTTGTGAGC-3′) to amplify alternative transcript products, and the reaction was run out on a 1% agarose gel in Tris-borate-EDTA buffer at 135 V for 1 h before imaging. Transcripts were quantified using a Luna Universal One-Step RT-qPCR Kit (NEB) and primers designed to target each individual transcript: F[+Exon13], 5′-GTTTGACATTGAGGCCTGGA-3′; F[–Exon13], 5′-CGACCCAGCAACCTGGAGAT-3′; F[Total pSPL3], 5′-AGGTGGAGAGAGAGACAGAG-3′; R[pSPL3], CCAGCCACCACCTTCTGATA-3′; F[*REEP5*], 5′-TGACGACACCTAGCTCTAAGAG-3′; R[*REEP5*], 5′-GGGAAGCCTCTTGTGTTTGA-3′. 500 ng RNA was used per reaction. qPCR was performed on the Eppendorf MasterCycler realplex2, and ΔΔCt values were calculated using *REEP5* as the housekeeping gene.

## Supplementary Material

10.1242/dmm.052690_sup1 Supplementary information
