# Supplementary material for: Multiple mechanisms lead to loss-of-function effects of pathogenic SARS2 variants
Source: Dis Model Mech. 2026 Jul 24;19(7):dmm052690. doi: 10.1242/dmm.052690 (PMC13446559; doi:10.1242/dmm.052690)
Supplement: Supplementary file 1 [file dmm-19-052690-s1.pdf]

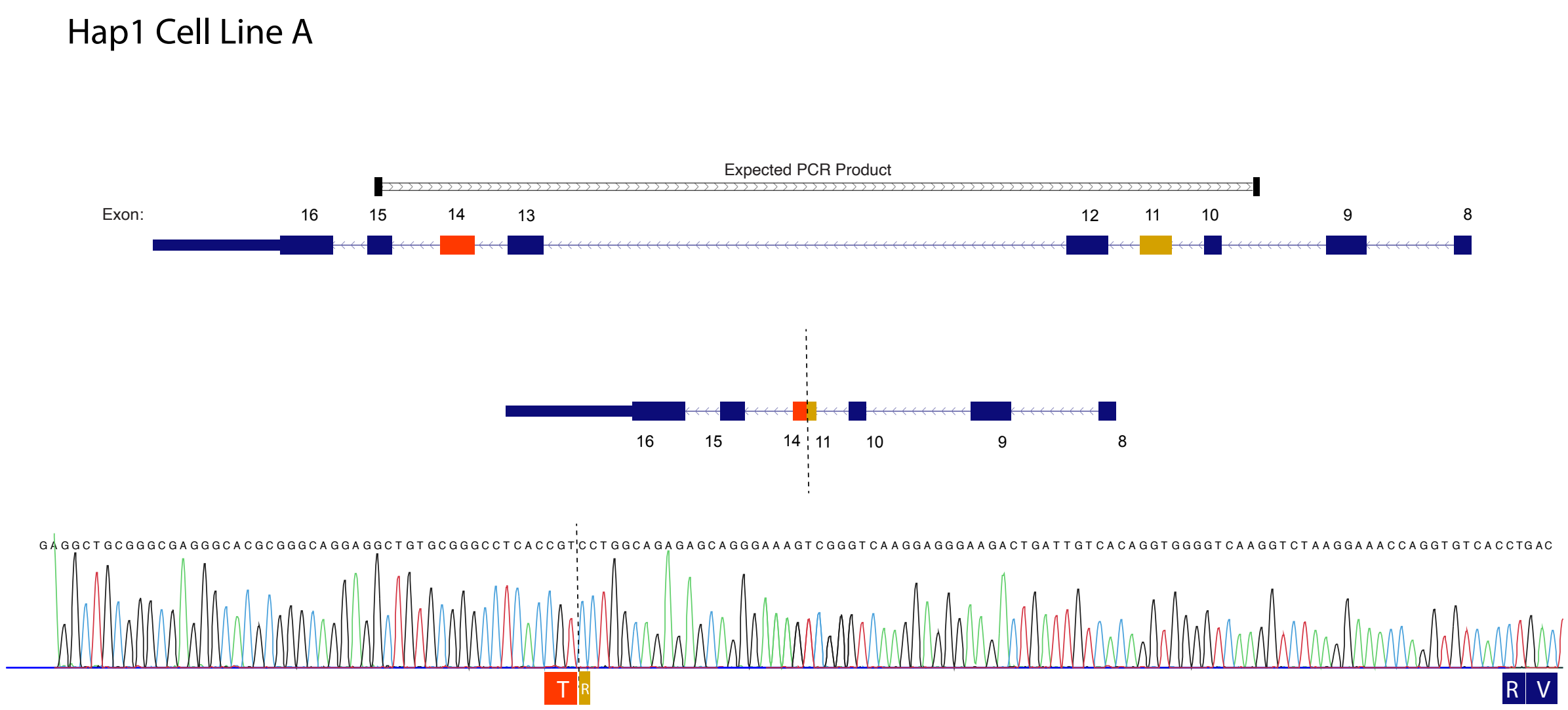

Fig. S1. Sanger sequencing of ΔSARS2+SARS2-1xFLAG cell line A. Sanger sequencing diagram showing the sequence of deleted nucleotides in cell line A.

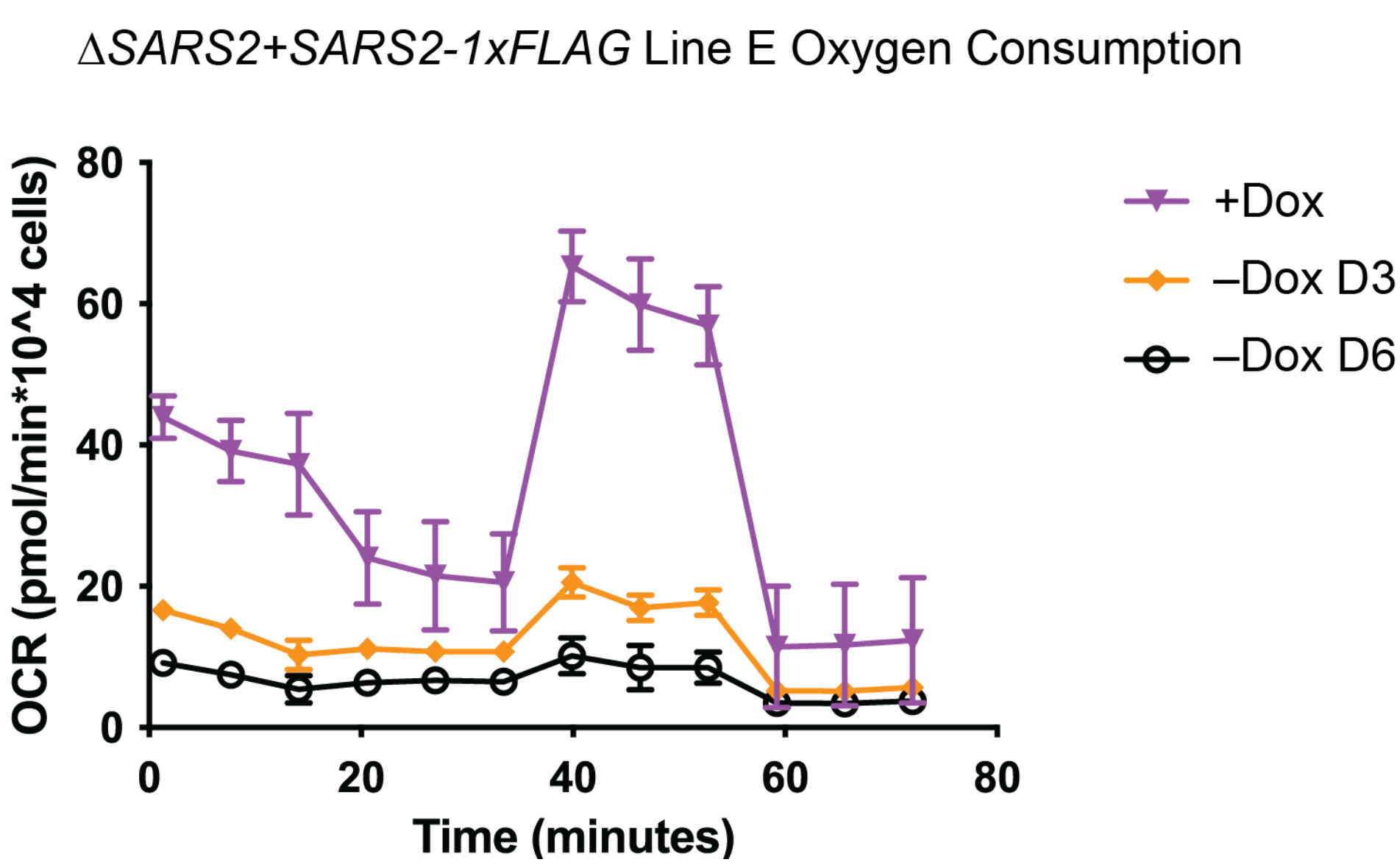

Fig. S2. Cell line E Seahorse validation. Seahorse mitochondrial stress test performed on cell line E grown with doxycycline or without doxycycline for 3 or 6 days.

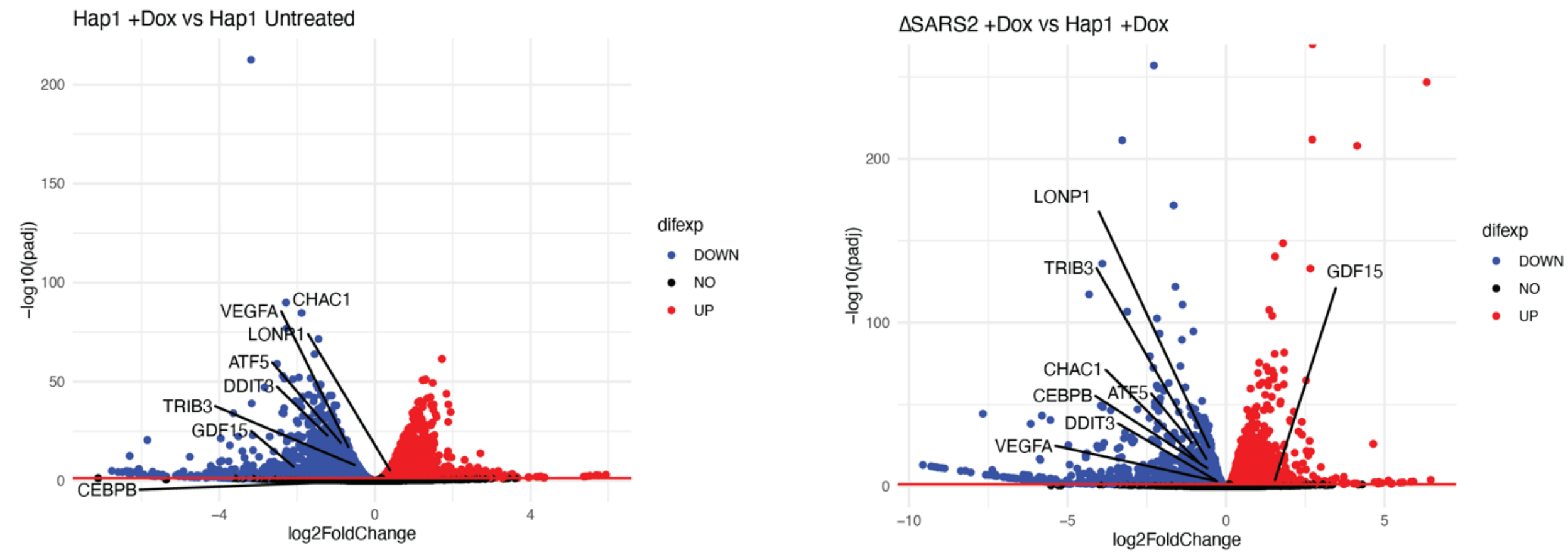

Fig. S3. Hap1 differential expression control analyses. Differential expression results of unmodified Hap1 cells grown with and without doxycycline to control for any differential expression effects caused by doxycycline treatment and/or genetic modification.

Disease Models & Mechanisms • Supplementary information

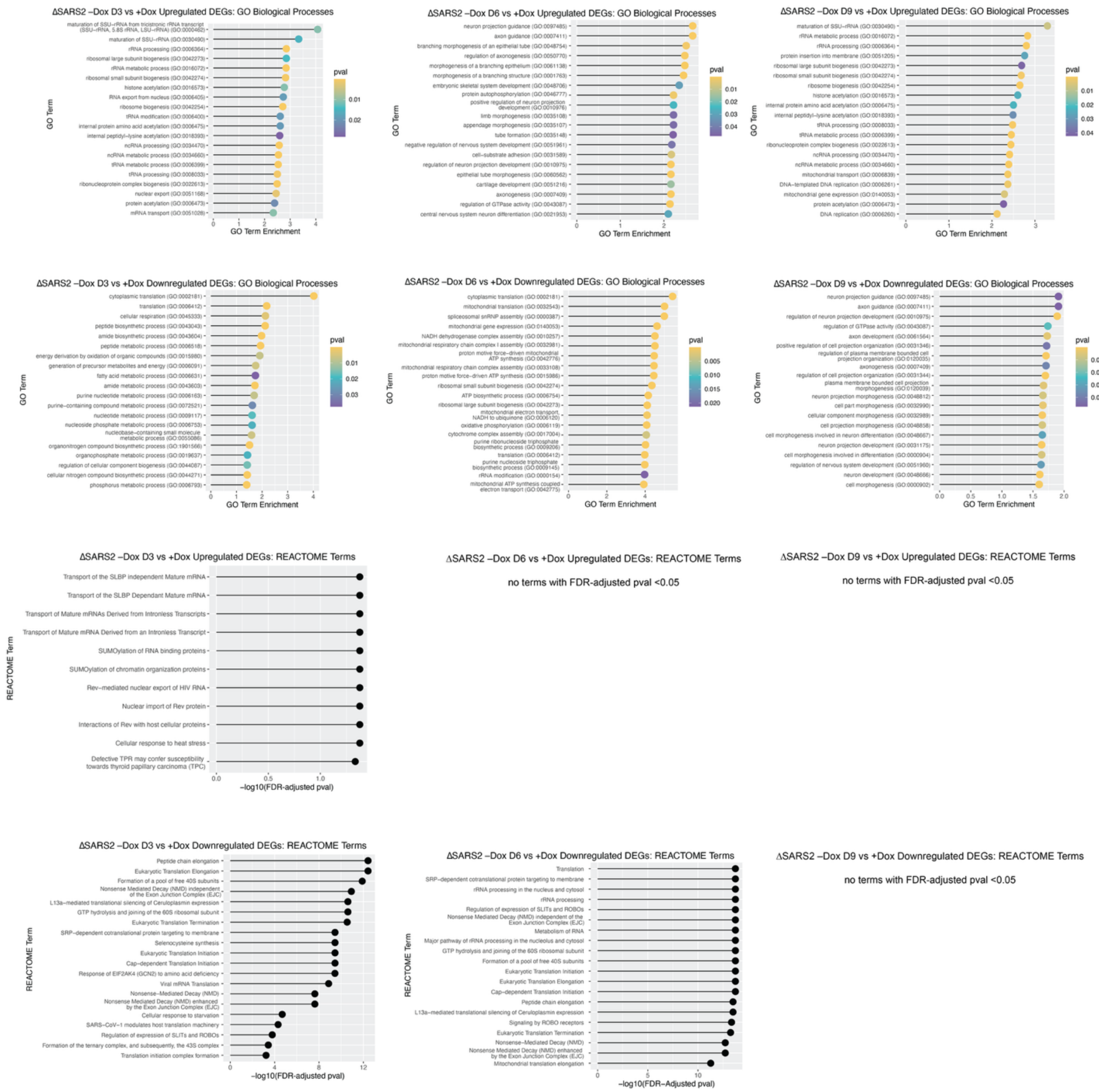

Fig. S4. Timepoint-specific GO term and REACTOME analyses. Analyses from Δ*SARS2*+*SARS2-1xFLAG* cells without doxycycline for 3, 6, and 9 days.

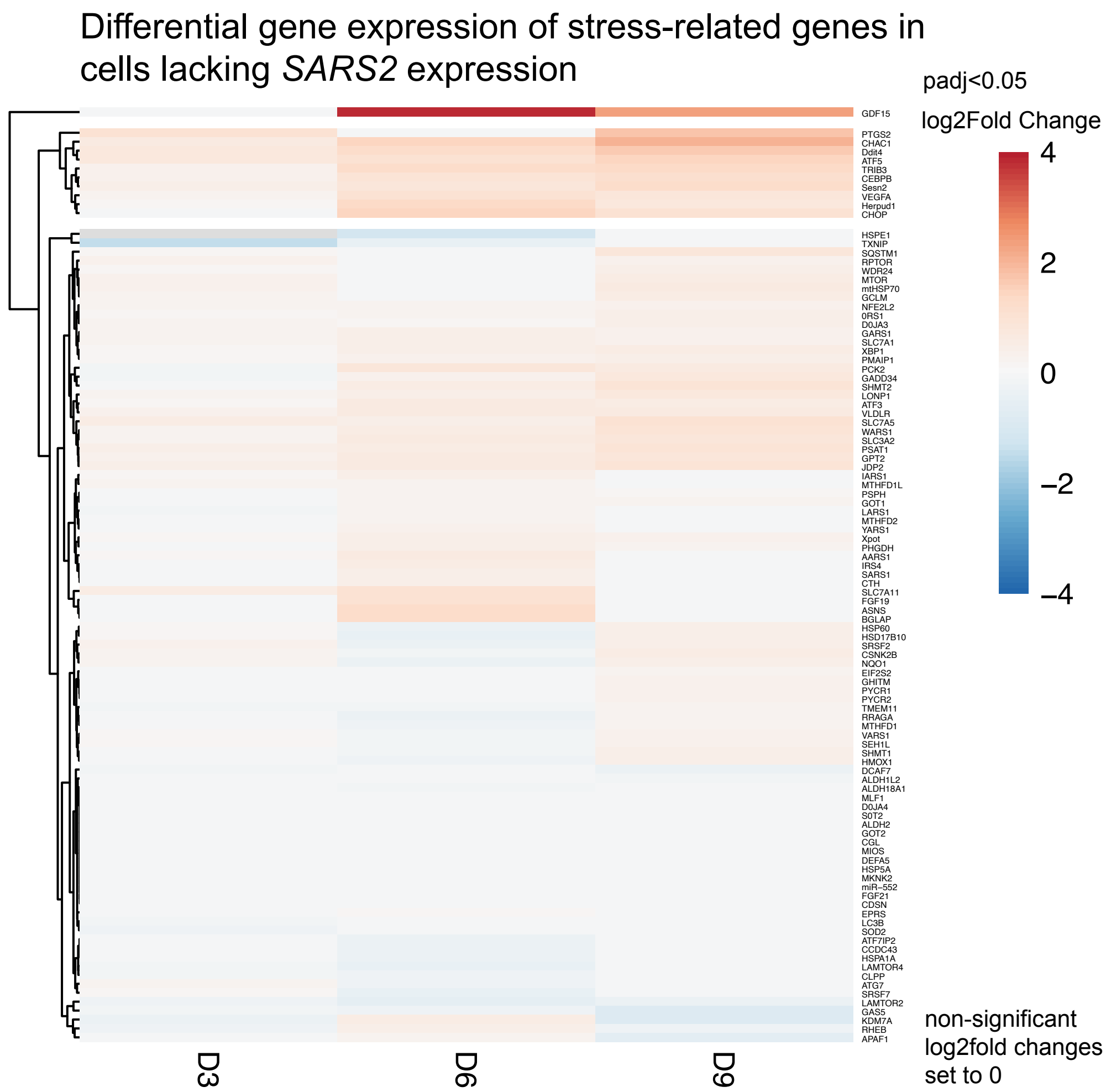

Fig. S5. Differential expression of stress-related genes. Heat map showing differential expression levels of manually-curated list of stress-related genes at each day without doxycycline compared to +doxycycline cells.

Disease Models & Mechanisms • Supplementary information

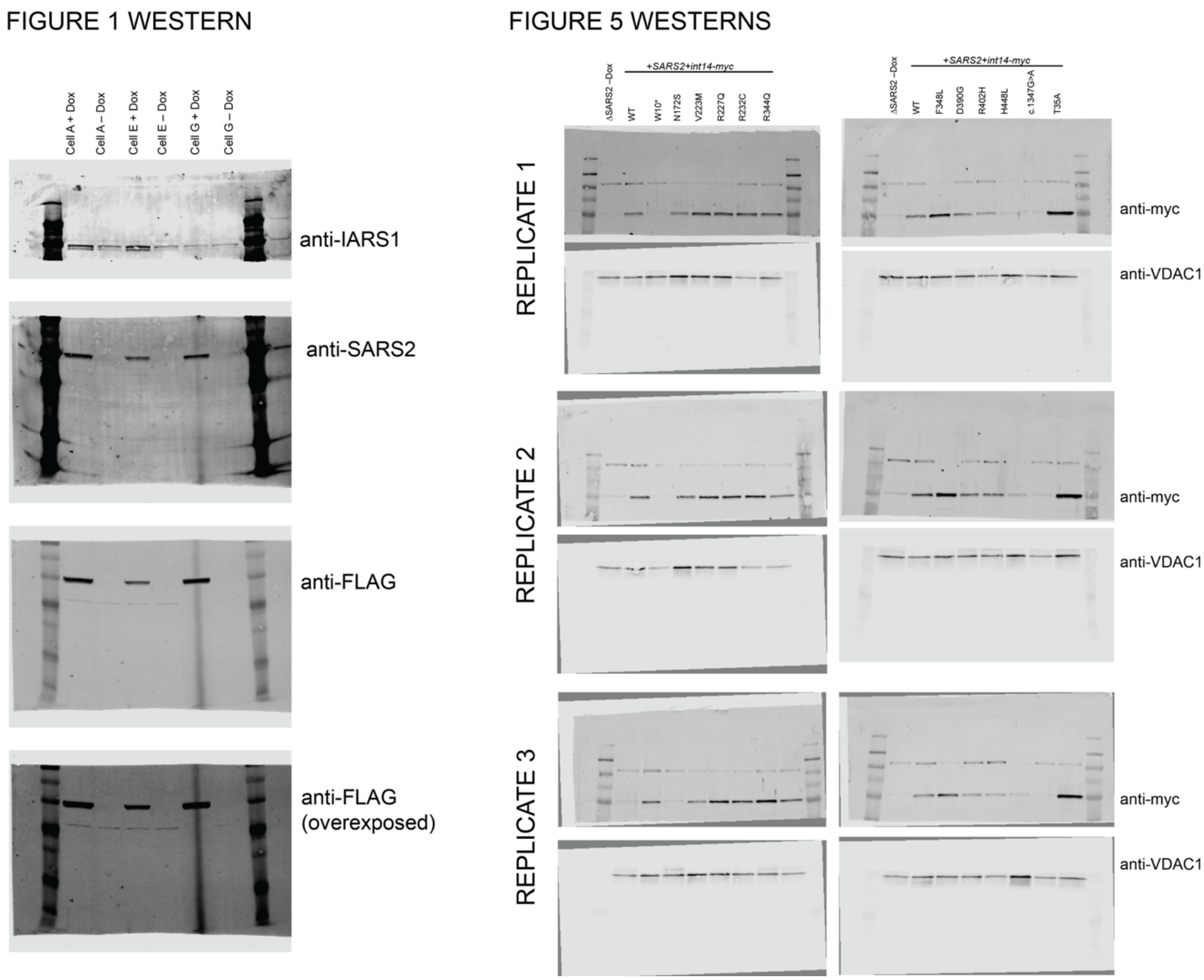

Fig. S6. Western blots. Full-length, untrimmed images for each western blot presented in the main text.

Disease Models & Mechanisms • Supplementary information

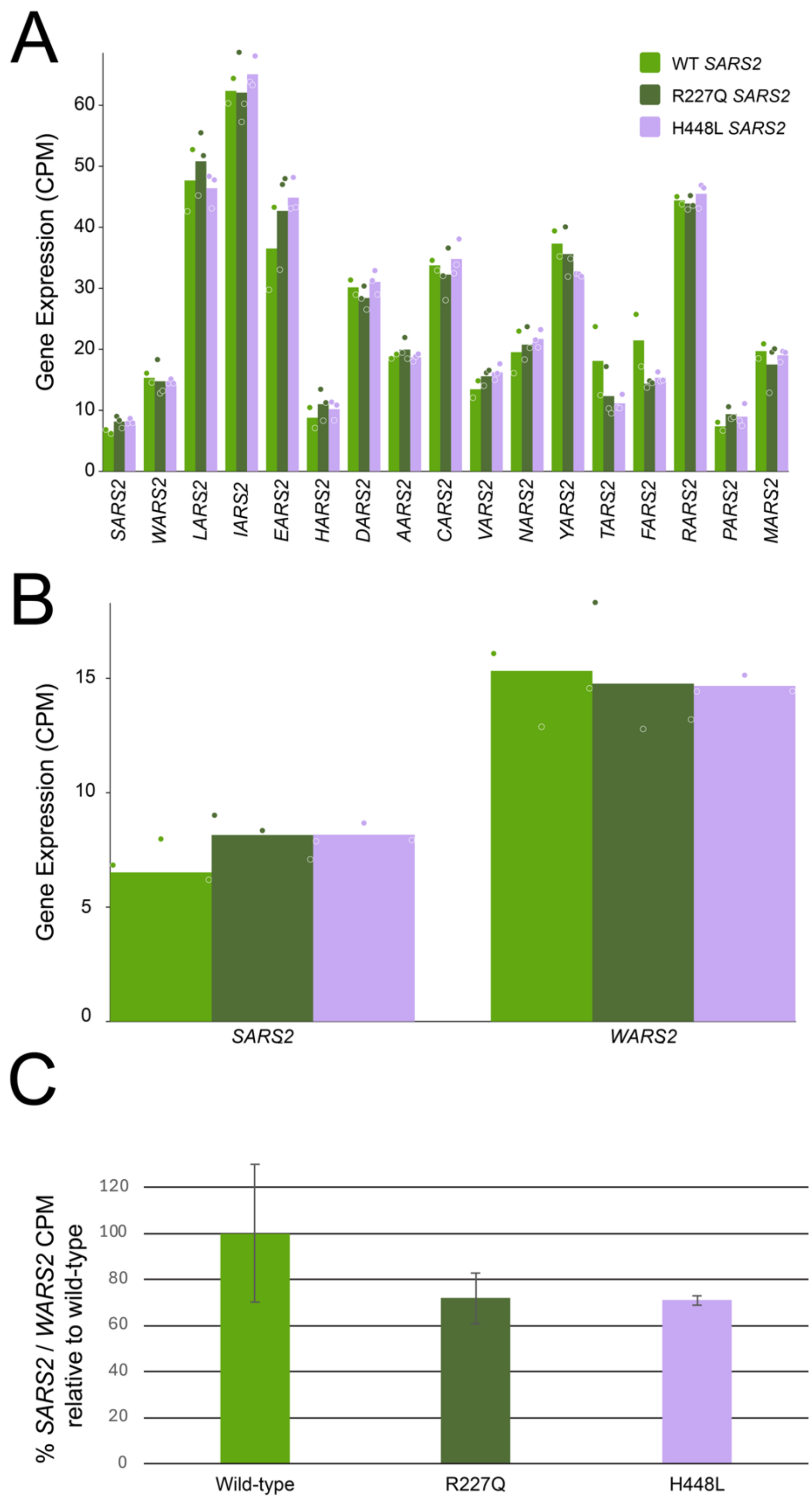

Disease Models & Mechanisms • Supplementary information

Fig. S7. RNA-seq expression analysis of non-clonal Hap1 cell populations expressing individual SARS2 alleles. (**A**) Gene expression analysis of all 17 mitochondrial ARS genes. Hap1 cells transduced with lentiviral constructs to express wild-type (light green), R227Q (dark green), or H448L (purple) *SARS2* were grown in the absence of doxycycline for six days, a timepoint where there is no detectable protein expression from the DOX-inducible wild-type *SARS2* transgene (**Fig. 2C**). Cells were harvested, RNA was isolated, and RNA-seq analysis was performed (Plasmidsaurus). Counts per million (CPM are indicated along the y-axis and each of the 17 mitochondrial ARS genes is indicated along the y-axis. (**B**) Same data from panel A but showing only *SARS2* and *WARS2* CPM data. (**C**) *SARS2* CPM data for each cell population (*i.e.*, those expressing wild-type, R227Q, or H448L *SARS2*, indicated along the bottom) were normalized to *WARS2* data and expressed relative to the ratio for the wild-type *SARS2*-expressing cell population.

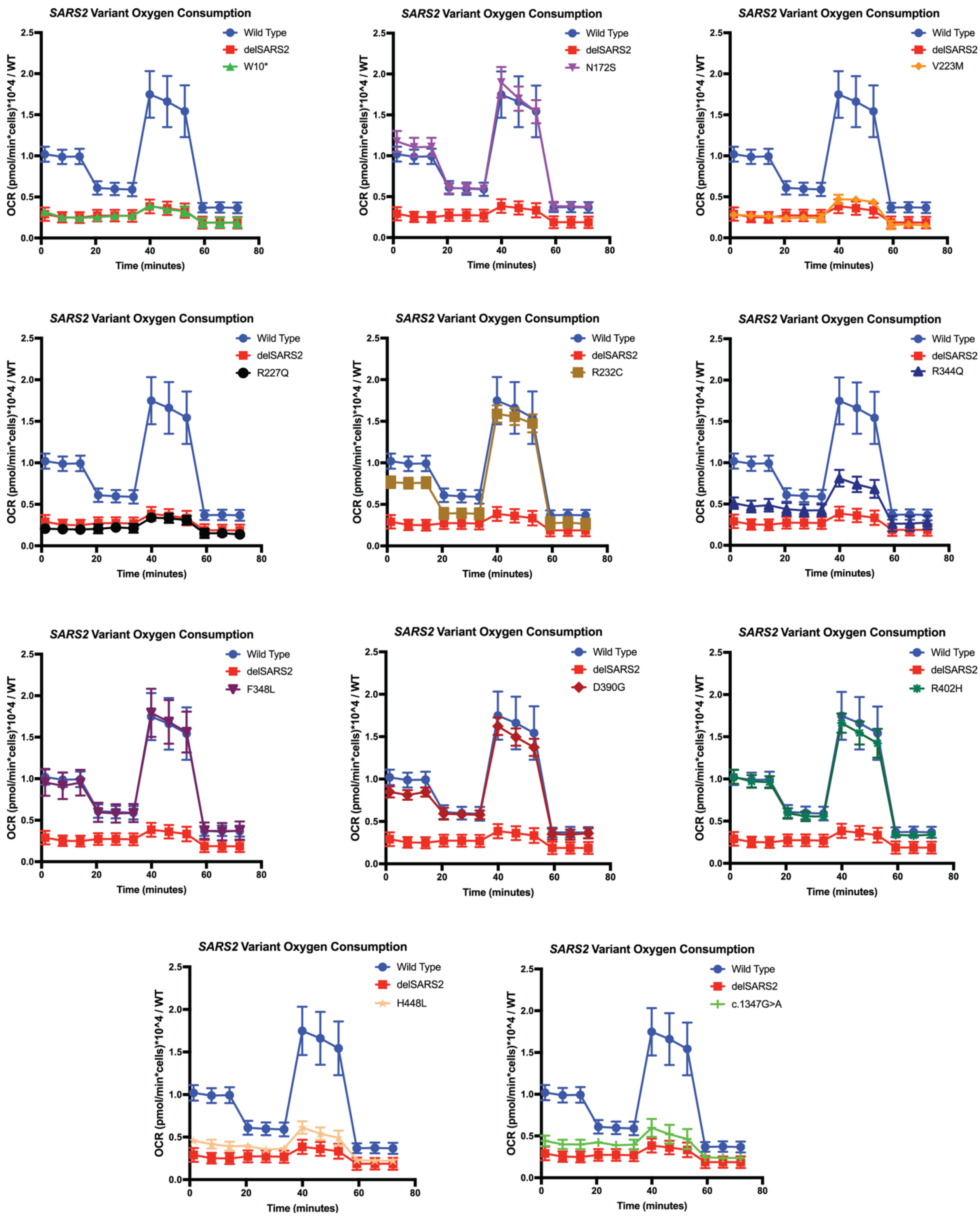

Fig. S8. Individual variant Seahorse graphs. Individual graphs with wild-type SARS2+int14-myc, untransduced Δ*SARS2*+*SARS2-1xFLAG*, and one pathogenic *SARS2* variant per panel.

Table S1. Primer sequences for mutagenic reactions

SARS2 N172S F	CGCTGAAGCTGCCCAGCCAGACCCACCCAGA
SARS2 N172S R	TCTGGGTGGGTCTGGCTGGGCAGCTTCAGCG
SARS2 V223M F	AAGCGCCTGTCCCACATGTCTGGCCACCGGT
SARS2 V223M R	ACCGGTGGCCAGACATGTGGGACAGGCGCTT
SARS2 R227Q F	ACGTGTCTGGCCACCAGTCCTATTACCTGCG
SARS2 R227Q R	CGCAGGTAATAGGACTGGTGGCCAGACACGT
SARS2 R232C F	CGGTCCTATTACCTGTGCGGGGCTGGAGCCC
SARS2 R232C R	GGGCTCCAGCCCCGCACAGGTAATAGGACCG
SARS2 R344Q F	CCCGGGGGCTGTATCAAGTACACCACTTCAC
SARS2 R344Q R	GTGAAGTGGTGTACTTGATACAGCCCCCGGG
SARS2 F348L F	TATCGAGTACACCACCTCACCAAGGTGGAGA
SARS2 F348L R	TCTCCACCTTGGTGAGGTGGTGTACTCGATA
SARS2 D390G F	ACTTCCGGGTCCTGGGTATGCCCACCCAAGA
SARS2 D390G R	TCTTGGGTGGGCATACCCAGGACCCGGAAGT
SARS2 H448L F	AGCTGCAGTTTGCCCTCACGGTGAGGCCCGC
SARS2 H448L R	GCGGGCCTCACCGTGAGGGCAAACTGCAGCT
SARS2 FLAG ins F	CCTGCTGTAAGCgactacaaagacgatgacgacaaaTAAGACCCAGCTTTC
SARS2 FLAG ins R	GAAAGCTGGGTCTTAtttgtcgtcatcgtctttgtagtcGCTTACAGCAGG

Please note that R402H and c.1347G>A *SARS2* constructs were synthesized commercially (Twist Biosciences).

Disease Models & Mechanisms • Supplementary information
